# Entropy‐Stabilized Aluminate Catalysts That Break the Activity–Stability Tradeoff in CF_4_ Hydrolysis

**DOI:** 10.1002/anie.6752036

**Published:** 2026-06-08

**Authors:** Seunghyuck Chi, Hyungmin Jeon, Yaejun Baik, DongHwan Oh, Jin Seok, Woosung Choi, Seungjun Lee, Minkee Choi

**Affiliations:** ^1^ Department of Chemical and Biomolecular Engineering (BK21 Four) Korea Advanced Institute of Science and Technology (KAIST) Daejeon Republic of Korea; ^2^ EHS/Infra Technology Research Center Samsung Electronics Co., Ltd Hwaseong Republic of Korea

**Keywords:** CF_4_ hydrolysis, entropy‐stabilized oxide, Lewis acid, Mars–van Krevelen, stability

## Abstract

Tetrafluoromethane (CF_4_) is a potent, long‐lived greenhouse gas widely used in semiconductor dry‐etching processes, yet its abatement via hydrolysis remains challenging due to the lack of catalysts that simultaneously exhibit high activity and durability under strongly fluorinating, steam‐rich conditions. Conventional alumina‐based catalysts suffer from severe activity–stability tradeoffs, offering good activity only at the cost of rapid deactivation through surface‐area loss, bulk fluorination, and the formation of catalytically inactive *α*‐Al_2_O_3_. Here, we report an entropy‐stabilized aluminate catalyst that overcomes this long‐standing tradeoff by combining a multication aluminate framework with an entropy‐stabilized lattice. The incorporation of multiple metal cations produces an electron‐deficient Al–O environment that enhances heterolytic C–F bond activation while suppressing H_2_O poisoning, thereby increasing intrinsic activity under practical conditions. Simultaneously, the lattice stabilization via high configurational entropy inhibits the propagation of fluorination into the bulk lattice, effectively suppressing in situ AlF_3_ formation and its subsequent hydrolysis to *α*‐Al_2_O_3_. Mechanistic studies further establish that CF_4_ hydrolysis proceeds via a Mars–van Krevelen‐type pathway in which lattice oxygen first oxidizes CF_x_ surface intermediates and is replenished by H_2_O. These findings identify entropy‐stabilized aluminates as a robust materials platform capable of simultaneously achieving high activity and long‐term durability for CF_4_ hydrolysis.

## Graphical abstract

Entropy‐stabilized aluminate breaks the activity–stability tradeoff in CF_4_ hydrolysis by combining a multication aluminate framework with an entropy‐stabilized lattice. An electron‐deficient Al–O environment is F‐philic but O‐phobic, enabling C–F activation while suppressing H_2_O poisoning. Under steam‐rich, strongly fluorinating conditions, the entropy‐stabilized lattice inhibits phase change and ripening, preserving high activity.

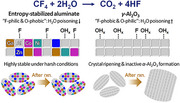

## 1 Introduction

Tetrafluoromethane (CF_4_) is a nonflammable, chemically inert perfluorocarbon widely used as a plasma etchant in semiconductor manufacturing [1]. Unfortunately, CF_4_ strongly absorbs infrared radiation and acts as a greenhouse gas with a global warming potential more than 6000 times that of CO_2_ [2]. Owing to its exceptionally strong C–F bonds (547 ± 2 kJ mol^−1^) [3], CF_4_ is highly stable and has an extraordinarily long atmospheric lifetime of ∼50000 years [2, 4]. These characteristics underscore the urgent need to decompose CF_4_ prior to its release into the atmosphere. Various abatement technologies, including plasma decomposition [5, 6, 7], combustion with fuel [8, 9, 10], and catalytic hydrolysis [11, 12, 13, 14, 15, 16, 17, 18, 19, 20, 21, 22, 23, 24, 25], have been extensively investigated. Plasma decomposition can achieve high removal efficiency, but it requires a large energy input and relies on complex, maintenance‐intensive equipment, making it costly and less attractive for large‐scale continuous operation. Combustion with fuel requires extremely high operating temperatures (> 2200 K) and a continuous fuel supply [10], leading to high energy consumption and secondary CO_2_ emissions. By contrast, catalytic hydrolysis (CF_4_ + 2H_2_O → CO_2_ + 4HF) is the most widely used method because it operates at lower temperatures (923–1123 K), consumes less energy, and enables continuous operation under industrial conditions [11].

In the catalytic hydrolysis of CF_4_, *γ*‐Al_2_O_3_‐based catalysts have been the most extensively studied and are widely employed in industry [11, 12, 13, 14]. *γ*‐Al_2_O_3_ provides abundant Lewis acid sites with strong chemical affinity for both F^–^ and H_2_O, enabling efficient C–F bond activation and subsequent hydrolysis. Among metal oxides, it is also relatively stable, abundant, cost‐effective, and can be readily shaped into mechanically robust forms. In industrial processes, a wet scrubber is typically used to pretreat the off‐gas (Figure S1); consequently, the H_2_O/CF_4_ molar ratio of the gas stream entering the catalytic converter is generally very high (> 50) [11, 12]. Despite its well‐known thermochemical stability, even *γ*‐Al_2_O_3_ undergoes gradual sintering and transforms into the catalytically inactive *α*‐Al_2_O_3_ phase when exposed to such excess H_2_O and HF product under harsh reaction conditions.

To enhance the structural stability of *γ*‐Al_2_O_3_, various dopants such as Zn [15, 16, 17, 18, 19, 20, 25], P [20, 21], Ga [17, 22, 24, 25], and Ni [17, 19, 23] have been incorporated into the oxide. In general, the introduction of these dopants decreases the surface area and lowers the density of Lewis acid sites compared to pristine *γ*‐Al_2_O_3_, often leading to diminished catalytic performance [17, 19]. Accordingly, such materials generally exhibit a characteristic activity–stability trade‐off in CF_4_ hydrolysis. Interestingly, despite the reduced Lewis acid density, *γ*‐Al_2_O_3_ with optimal Zn loadings exhibits higher catalytic activity than pristine *γ*‐Al_2_O_3_, and Zn is thus frequently cited as one of the most effective dopants [17, 19]. Our previous studies revealed that controlled Zn addition generates a ZnAl_2_O_4_ overlayer on the *γ*‐Al_2_O_3_ surface, which suppresses the *γ*→*α* phase transformation [18]. Moreover, this ZnAl_2_O_4_ layer adsorbs CF_4_ more effectively under excess H_2_O, mitigating H_2_O‐induced surface poisoning and thereby enhancing overall catalytic performance. Nevertheless, even these doped *γ*‐Al_2_O_3_ materials gradually deactivate under harsh reaction conditions, underscoring the need for a new class of materials with substantially improved structural stability.

Entropy‐stabilized oxides are a class of materials synthesized by incorporating multiple metal cations into a single‐phase oxide lattice [26, 27]. The presence of numerous distinct cations increases the configurational entropy; according to the Gibbs free energy relation ∆*G* = ∆*H*—*T*∆*S*, the *T*∆*S* term can become sufficiently large at elevated temperatures to stabilize single‐phase solid solutions rather than multiphase mixtures [28]. Consequently, entropy‐stabilized oxides represent oxide systems in which cation‐site disorder helps maintain structural integrity under harsh conditions [26, 29, 30]. This entropy‐driven stabilization can provide exceptional resistance to phase transitions and chemical degradation under corrosive reaction environments, making entropy‐stabilized oxides particularly promising as stable catalysts for CF_4_ hydrolysis. Here, we introduce an entropy‐stabilized aluminate (ESA) designed to overcome the long‐standing activity–stability tradeoff of *γ*‐Al_2_O_3_‐based catalysts. By integrating multiple metal cations into a single spinel framework, ESA exhibits high activity, suppressed H_2_O poisoning, and long‐term stability under H_2_O‐rich, fluorinating reaction conditions.

## 2 Results and Discussion

### 2.1 Structural Analysis of Catalysts

The ESA formulation was established based on heterometal compatibility with the aluminate spinel lattice and an Al‐rich composition required for spinel formation. The heterometal cations were selected by considering the spinel tolerance factor (*τ*), a geometric descriptor derived from ionic radii for assessing spinel phase stability [31]. Reported spinel compounds are typically distributed around *τ* ≈ 0.85; therefore, cations with *τ* values in the range of approximately 0.84–0.90 (e.g., Zn, Ga, Ni, Co, and Mg) were considered as structurally viable candidates (Table S1 and Supplementary Note 1). Among these, Mg was excluded because its oxide is basic and may not favor the Lewis acidic Al–O environment required for CF_4_ activation. The Al:Zn:Ga:Ni:Co molar ratio was fixed at 8:1:1:1:1 (Table 1). The Al‐rich composition was chosen to satisfy the structural requirements of the spinel lattice, for which a high fraction of Al^3+^ is required to stabilize the octahedral sublattice while enabling multication incorporation on both tetrahedral and octahedral sites. The resulting configurational entropy (Δ*S*_conf_), calculated from the overall cation distribution, is approximately 1.1R (Supplementary Note 2), a value comparable to those reported for entropy‐stabilized spinel and perovskite oxide systems [32, 33, 34, 35]. Thus, this formulation serves as a model system for evaluating multication stabilization effects while preserving the catalytically relevant Al–O framework. Based on this formulation, ESA was synthesized by hydrothermally treating pseudoboehmite in an aqueous solution containing Zn(NO_3_)_2_, Ga(NO_3_)_3_, Ni(NO_3_)_2_, and Co(NO_3_)_2_ at 443 K, followed by calcination at 1073 K.

**TABLE 1:** Physicochemical properties of catalysts.

Sample	*S*_BET_ ^a^ (m^2^ g^−1^)	*V*_p_ ^b^ (cm^3^ g^−1^)	*D*_p_ ^c^ (nm)	*n*_Lewis_ ^d^ (µmol g^−1^)	Zn/Al^e^	Ga/Al^e^	Ni/Al^e^	Co/Al^e^
ESA	101	0.21	9.6	133	0.13	0.12	0.12	0.12
ESA–Zn	89	0.14	10.4	92	N/A	0.14	0.17	0.15
ESA–Ga	68	0.19	15.4	80	0.17	N/A	0.16	0.17
ESA–Ni	74	0.13	11.6	89	0.17	0.16	N/A	0.17
ESA–Co	100	0.18	10.9	107	0.17	0.18	0.18	N/A
*γ*‐Al_2_O_3_	178	0.32	11.6	210	N/A	N/A	N/A	N/A
ZnAl_2_O_4_@*γ*‐Al_2_O_3_	143	0.26	12.1	175	0.11	N/A	N/A	N/A

^a^ BET surface areas calculated from N_2_ adsorption isotherms in the *P*/*P*_0_ range of 0.05–0.15.

^b^ Total pore volumes determined at *P*/*P*_0_ = 0.95.

^c^ Pore sizes calculated using nonlocal density functional theory (NLDFT) analysis.

^d^ Lewis acid densities determined by FT‐IR spectroscopy after pyridine adsorption at 423 K.

^e^ Atomic ratios determined by ICP‐OES.

**FIGURE 1:**
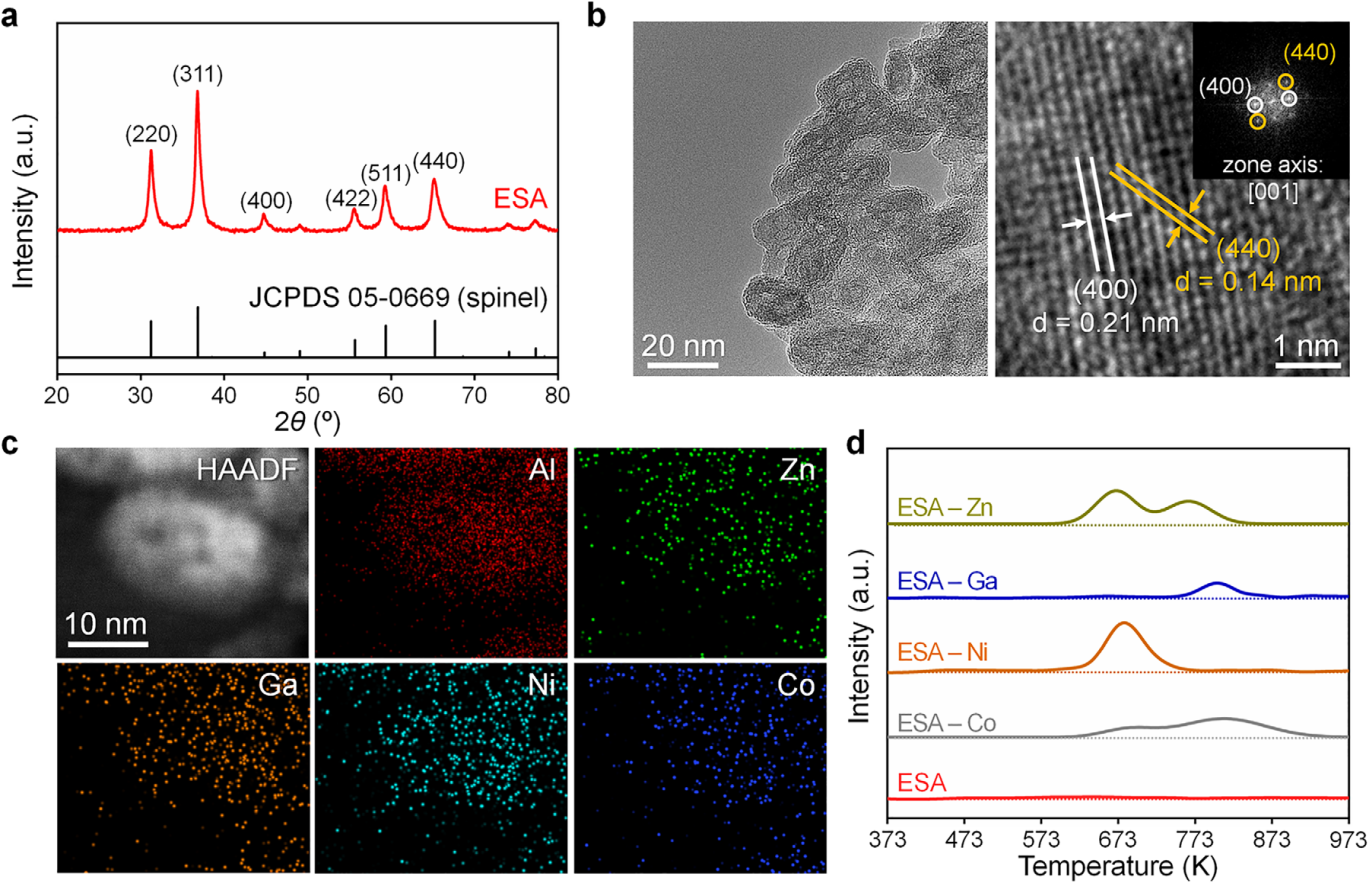
(a) XRD pattern of ESA and the reference pattern of spinel ZnAl_2_O_4_ (JCPDS card no. 05–0669). (b) TEM images of ESA (inset: fast Fourier transform pattern). (c) HAADF‐STEM and EDS elemental mapping images of ESA. (d) H_2_‐TPR profiles of ESA and mixed oxides obtained by removing one of the constituent elements (Zn, Ga, Ni, or Co) from ESA (ESA–*elem*. samples).

The X‐ray diffraction (XRD) pattern of ESA displayed reflections corresponding exclusively to a single spinel phase, with no detectable impurity phases (Figure 1a). Transmission electron microscopy (TEM) also revealed 10–20 nm nanocrystallites exhibiting lattice fringes with d‐spacings characteristic of a spinel structure (Figure 1b). High‐angle annular dark‐field scanning transmission electron microscopy (HAADF‐STEM) and elemental mapping by energy dispersive spectroscopy (EDS) at high (Figure 1c) and low (Figure S2) magnifications confirmed a uniform spatial distribution of all constituent elements both across and within individual crystallites. Furthermore, the elemental composition determined by EDS and inductively coupled plasma optical emission spectrometry (ICP‐OES) closely matched the nominal atomic ratios (Al:Zn:Ga:Ni:Co = 8:1:1:1:1), thereby verifying homogeneous multication incorporation throughout the crystallites (Table 1 and Table S2).

To evaluate the role of configurational entropy, we additionally prepared mixed oxides in which one of the elements (Zn, Ga, Ni, or Co) was individually removed from the ESA formulation (Table 1). The samples were designated as “ESA—*elem*.,” where *elem*. denotes the omitted element. For example, “ESA—Zn” refers to the sample synthesized without Zn in the ESA composition. EDS elemental mapping revealed pronounced elemental zoning in all of these samples, indicative of phase separation (Figure S3). The segregated domains were primarily enriched in Ni and Co. Consistently, XRD analysis showed additional reflections assignable to a rock‐salt phase, consistent with the crystal structures of NiO and CoO (Figure S4). These observations indicate that NiO‐ and CoO‐rich domains readily segregate when configurational entropy is reduced by the removal of a constituent element. This behavior aligns with established phase‐formation trends. Ni^2+^ and Co^2+^ typically form NiAl_2_O_4_ and CoAl_2_O_4_ spinels only at very high temperatures (> 1173 K) [36, 37, 38], as their incorporation into the alumina lattice demands extensive cation diffusion and is further hindered by their octahedral site preference [39]. In contrast, Zn^2+^ readily occupies tetrahedral sites, enabling the formation of ZnAl_2_O_4_ spinel at lower temperatures (∼973 K) [37], whereas Ga^3+^ does not form a distinct spinel phase but can substitute for Al^3+^ in the *γ*‐Al_2_O_3_ lattice due to their similar ionic radii [40].

To provide additional evidence for entropy‐assisted stabilization, we conducted in situ high‐temperature XRD measurements for ESA and ESA–Ga in the temperature range of 773–1073 K (Figure S5). ESA–Ga showed progressively increasing rock‐salt reflections above 873 K. In contrast, ESA maintained a single spinel phase without detectable phase separation over the entire temperature range. Temperature‐programmed reduction (H_2_‐TPR) further underscored the importance of configurational entropy in stabilizing the multication framework (Figure 1d). ESA exhibited no reduction features up to 973 K, indicating that even the readily reducible Ni^2+^ and Co^2+^ ions are strongly stabilized within the entropy‐stabilized matrix. In contrast, distinct reduction peaks were observed in the ESA–*elem*. samples. These results demonstrate that insufficient configurational entropy leads to metal‐oxide domain segregation and increased chemical lability of the cations.

To benchmark the catalytic performance in CF_4_ hydrolysis, *γ*‐Al_2_O_3_ and Zn‐promoted *γ*‐Al_2_O_3_ (denoted as “ZnAl_2_O_4_@*γ*‐Al_2_O_3_”), which are widely used in industrial practice [11, 12, 13, 14], were also synthesized. ZnAl_2_O_4_@*γ*‐Al_2_O_3_ was prepared with an atomic Zn/Al ratio of 0.1 [18], as verified by ICP‐OES (Table 1). This Zn loading corresponds to approximately 20% of that required for complete conversion of *γ*‐Al_2_O_3_ to ZnAl_2_O_4_. XRD analysis showed broad reflections characteristic of defective spinel‐like *γ*‐Al_2_O_3_, while ZnAl_2_O_4_@*γ*‐Al_2_O_3_ additionally exhibited crystalline ZnAl_2_O_4_ spinel peaks (Figure S6). TEM images revealed similar needle‐like crystal morphologies in both samples (Figure S7). However, EDS mapping of ZnAl_2_O_4_@*γ*‐Al_2_O_3_ showed pronounced Zn enrichment in the outer region of the crystallites (Figure S8), indicating the formation of a core–shell‐like architecture in which nanocrystalline ZnAl_2_O_4_ grows on the *γ*‐Al_2_O_3_ surface. This observation is consistent with our previous findings [18].

All catalyst samples (ESA, ESA—*elem*., *γ*‐Al_2_O_3_, and ZnAl_2_O_4_@*γ*‐Al_2_O_3_) exhibited type IV N_2_ adsorption–desorption isotherms, characteristic of mesoporous materials (Figure S9). As shown in the TEM images (Figure 1b, Figure S7), this mesoporosity originates from the intercrystalline voids formed among the oxide nanocrystallites. The mesopore size distributions of the samples fell within the range of 9.6–15.4 nm (Figure S10). The Brunauer–Emmett–Teller (BET) surface areas generally decreased with increasing heterometal incorporation or with more extensive formation of the spinel phase. Consequently, *γ*‐Al_2_O_3_ exhibited the highest surface area (178 m^2^ g^−1^), followed by ZnAl_2_O_4_@*γ*‐Al_2_O_3_ (143 m^2^ g^−1^), whereas both ESA and the ESA–*elem*. samples displayed substantially lower surface areas (Table 1). Interestingly, despite enhanced heterometal incorporation and more efficient spinel formation, ESA exhibited a higher surface area (101 m^2^ g^−1^) than the ESA–*elem*. samples (68–100 m^2^ g^−1^). This behavior is attributed to extensive multication mixing, which induces significant lattice distortion during oxide crystallization. This structural disorder has been reported to enhance nucleation while suppressing crystallite growth, ultimately yielding smaller oxide domains and a higher surface area [41]. Fourier transform infrared (FT‐IR) spectroscopy after pyridine adsorption (Figure S11) showed only bands associated with pyridine bound to Lewis acid sites for all samples, confirming the exclusive presence of Lewis acidity. The mass‐normalized density of Lewis acid sites was found to increase with increasing BET surface area of the samples (Table 1).

### 2.2 CF_4_ Hydrolysis Properties of Catalysts

**FIGURE 2:**
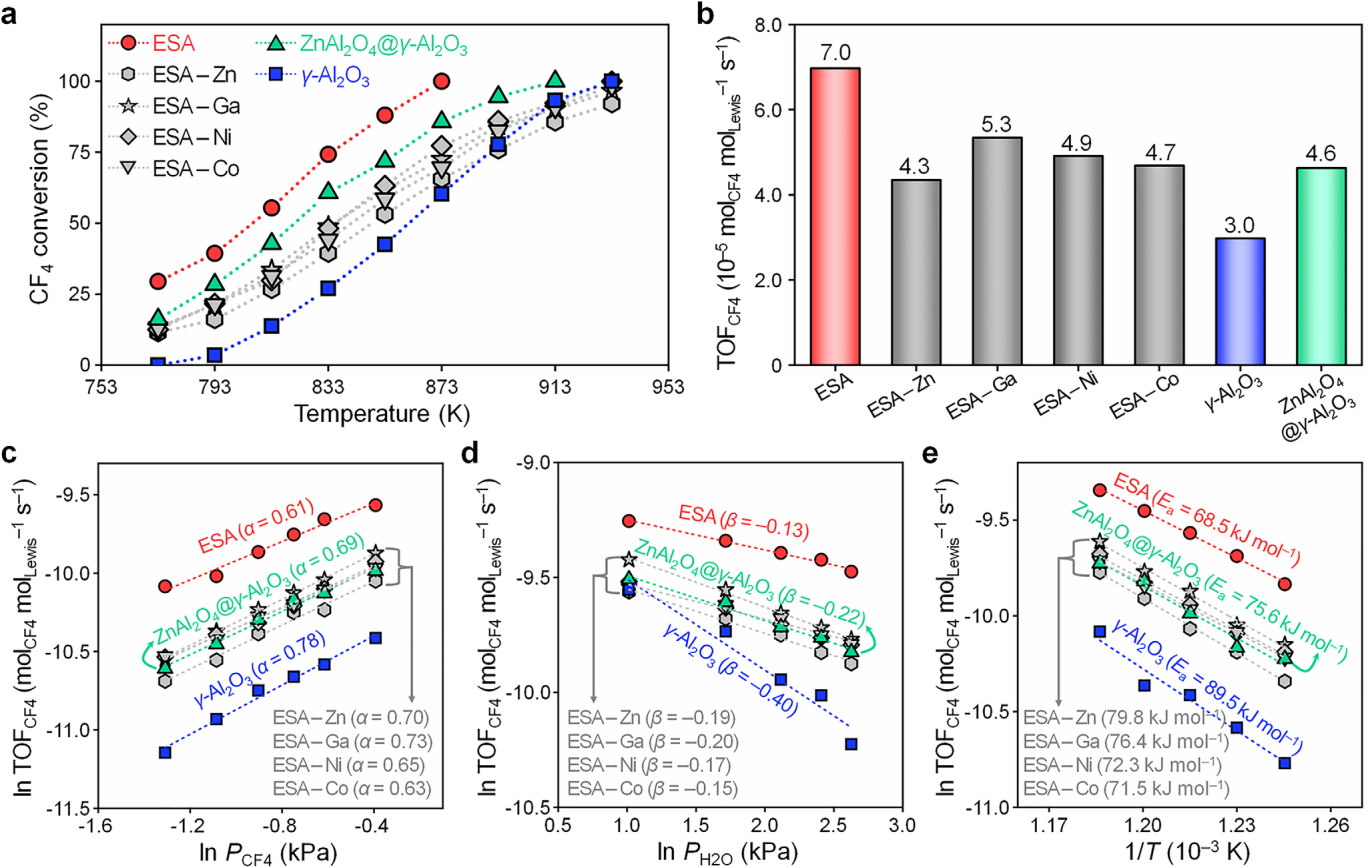
(a) CF_4_ conversion as a function of reaction temperature in CF_4_ hydrolysis (reaction conditions: 0.25 kPa CF_4_, 0.25 kPa Ar, and 12.7 kPa H_2_O in He balance; CF_4_‐based WHSV = 0.01 h^−1^). (b) Turnover frequency for CF_4_ conversion (TOF_CF4_) at 823 K. (c) Reaction order with respect to CF_4_ partial pressure (*α*) at 823 K. (d) Reaction order with respect to H_2_O partial pressure (*β*) at 823 K. (e) Arrhenius plots and apparent activation energies (*E*_a_).

The catalytic performances of the prepared materials were evaluated for CF_4_ hydrolysis at a fixed H_2_O/CF_4_ molar ratio of 50 under a CF_4_‐based weight hourly space velocity (WHSV) of 0.01 h^−1^ (Figure 2a), comparable to those used in previous studies [17, 18, 19, 20, 21, 22, 24, 25]. ESA exhibited the highest activity across the entire temperature range and consequently reached complete conversion at a lower temperature than the other catalysts. Notably, ESA outperformed conventional *γ*‐Al_2_O_3_ as well as ZnAl_2_O_4_@*γ*‐Al_2_O_3_, which was the best‐performing catalyst in our previous work [18], despite its lower Lewis acid site density (Table 1).

Since CF_4_ activation occurs exclusively on Lewis acidic Al centers, turnover frequency (TOF_CF4_) normalized to Lewis site density provides an appropriate measure of intrinsic site activity. To assess intrinsic activity, TOF_CF4_ was determined at 823 K under low‐conversion conditions (< 20%) to ensure kinetic relevance (Figure 2b). The TOF_CF4_ of ESA (7.0 × 10–^5^ mol_CF4_ mol_Lewis_^−1^ s^−1^) was approximately 2.3 and 1.5 times higher than those of *γ*‐Al_2_O_3_ (3.0 × 10^−5^ mol_CF4_ mol_Lewis_^−1^ s^−1^) and ZnAl_2_O_4_@*γ*‐Al_2_O_3_ (4.6 × 10^−5^ mol_CF4_ mol_Lewis_^−1^ s^−1^), respectively. The ESA–*elem*. samples exhibited TOF_CF4_ values (4.3–5.3 × 10^−5^ mol_CF4_ mol_Lewis_^−1^ s^−1^) comparable to that of ZnAl_2_O_4_@*γ*‐Al_2_O_3_ (Figure 2b).

All catalysts exhibited a positive reaction order with respect to CF_4_ partial pressure (Figure 2c) and a negative order with respect to H_2_O (Figure 2d), indicating that H_2_O adsorption dominates the catalyst surfaces (i.e., H_2_O poisoning). This H_2_O poisoning behavior is consistent with previous kinetic studies [18]. Notably, ESA showed a lower CF_4_ reaction order (0.61) than *γ*‐Al_2_O_3_ (0.78), ZnAl_2_O_4_@*γ*‐Al_2_O_3_ (0.69), and ESA–*elem*. samples (0.63–0.73), while also exhibiting a less negative H_2_O order (–0.13) compared with *γ*‐Al_2_O_3_ (–0.40), ZnAl_2_O_4_@*γ*‐Al_2_O_3_ (–0.22), and the ESA–*elem*. samples (–0.20 to –0.15). These trends indicate that ESA experiences mitigated H_2_O poisoning relative to the other catalysts.

Arrhenius analysis revealed that ESA exhibits a significantly lower apparent activation barrier (*E*_a_ = 68.5 kJ mol^−1^) than *γ*‐Al_2_O_3_ (89.5 kJ mol^−1^), ZnAl_2_O_4_@*γ*‐Al_2_O_3_ (75.6 kJ mol^−1^), and the ESA–*elem*. samples (71.5–79.8 kJ mol^−1^) (Figure 2e). Considering that the apparent activation barrier primarily reflects the rate‐determining C–F bond activation step [42, 43], these results suggest that ESA can more readily activate the exceptionally strong C–F bond in CF_4_. Collectively, the kinetic data demonstrate that the superior activity of ESA arises from its ability to alleviate H_2_O poisoning and promote C–F bond activation.

### 2.3 Chemisorption Properties of CF_4_ and H_2_O

**FIGURE 3:**
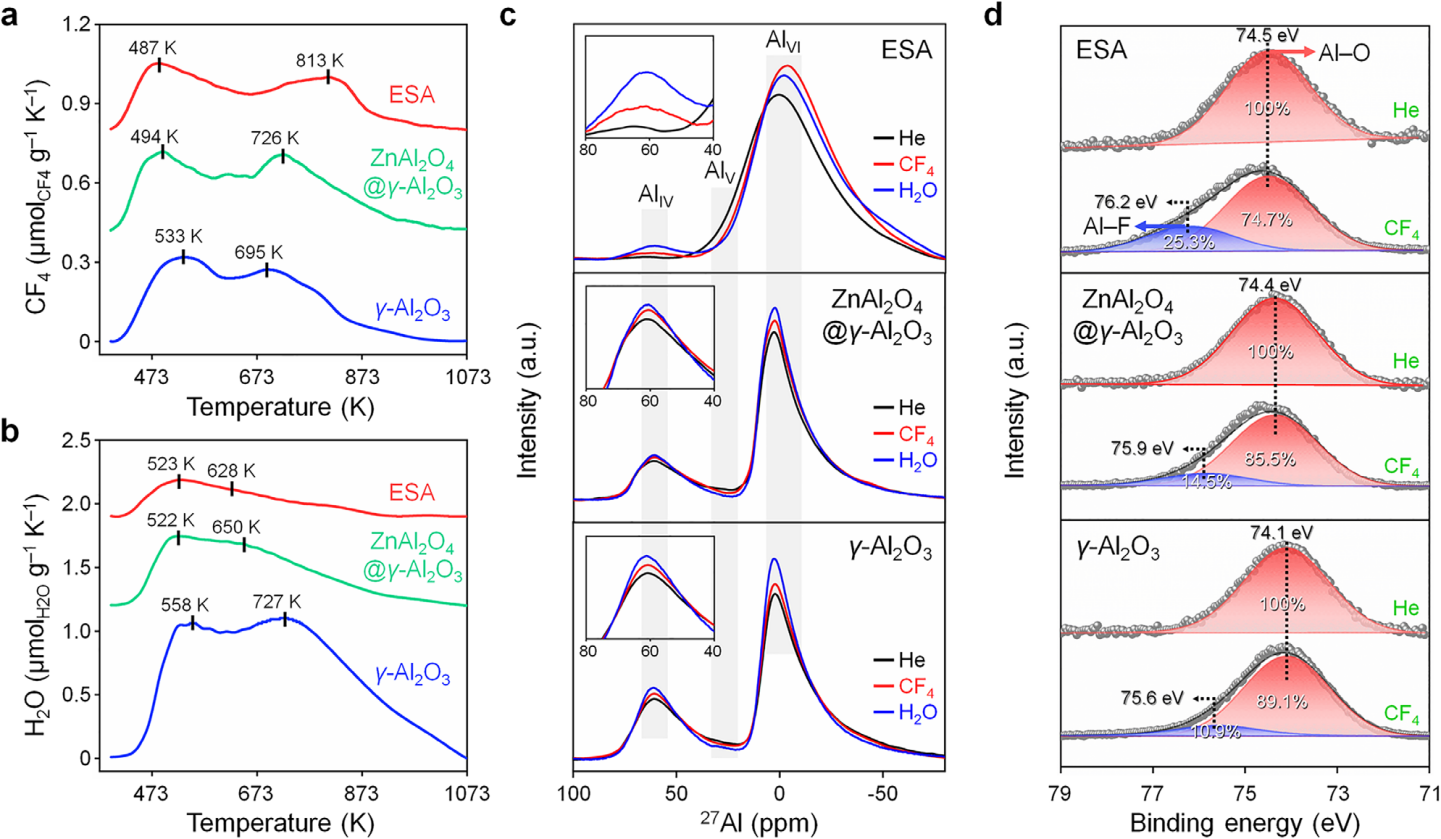
(a) CF_4_ and (b) H_2_O TPD–MS profiles of the catalysts. (c) Solid‐state ^27^Al MAS NMR spectra after He (black), CF_4_ (red), and H_2_O (blue) treatments at 873 K. (d) Al 2p XPS spectra after He and CF_4_ treatment at 873 K.

The surface properties of three representative catalysts, ESA, *γ*‐Al_2_O_3_, and ZnAl_2_O_4_@*γ*‐Al_2_O_3_, were systematically examined using complementary techniques. Temperature‐programmed desorption mass spectrometry (TPD–MS) profiles of the reactants, CF_4_ and H_2_O, were collected after pretreating the samples at 873 K and cooling to 373 K in each gas atmosphere. In the CF_4_ TPD–MS profiles (Figure 3a), two well‐resolved desorption peaks were observed for all catalysts: a low‐temperature peak at 480–540 K and a high‐temperature peak at > 600 K. Because the high‐temperature peak disappeared when the samples were pretreated mildly at 373 K (i.e., without the 873 K pretreatment) (Figure S12), it was assigned to desorption of strongly chemisorbed (dissociatively adsorbed) CF_4_. In contrast, the low‐temperature peak was attributed to the desorption of weakly physisorbed CF_4_. Since chemisorbed CF_4_ species play a key role in CF_4_ hydrolysis, comparison of the high‐temperature desorption features provides valuable insight into catalyst performance. The TPD–MS profiles showed that the center of the high‐temperature desorption peak shifted to progressively higher temperatures in the order *γ*‐Al_2_O_3_ (695 K) < ZnAl_2_O_4_@*γ*‐Al_2_O_3_ (726 K) < ESA (813 K). The high desorption temperature observed for ESA indicates stronger chemisorption of CF_4_, consistent with its more efficient C–F bond activation.

In the H_2_O TPD–MS profiles (Figure 3b), two desorption peaks were similarly observed at low temperature (500–600 K) and high temperature (> 600 K). The low‐temperature peak was attributed to the desorption of weakly adsorbed H_2_O, arising from hydrogen‐bonded surface species, whereas the high‐temperature peak was assigned to the recombinative desorption of strongly bound surface hydroxyl groups (i.e., chemisorbed H_2_O formed via Al hydroxylation) [44]. The catalytically relevant high‐temperature peak increased in intensity and shifted to a higher temperature in the order ESA (628 K) < ZnAl_2_O_4_@*γ*‐Al_2_O_3_ (650 K) < *γ*‐Al_2_O_3_ (727 K). Notably, this trend is the inverse of that observed in the CF_4_ TPD–MS results. These observations indicate that ESA exhibits the most efficient chemisorption of CF_4_ (i.e., C–F bond activation) while displaying the weakest H_2_O chemisorption among the catalysts. These surface properties rationalize why ESA shows the lowest apparent activation barrier (*E*_a_), consistent with the most efficient activation of the stable C–F bond, and the least negative H_2_O reaction order, reflecting its highly suppressed H_2_O poisoning.

Solid‐state ^27^Al magic‐angle spinning nuclear magnetic resonance (^27^Al MAS NMR) spectroscopy was used to probe Al coordination environments and their changes upon CF_4_ or H_2_O exposure at 873 K (Figure 3c). In the He‐treated pristine samples, three resonances were observed at approximately 60, 30, and 0 ppm, corresponding to tetrahedral, pentacoordinate, and octahedral Al environments, respectively [45, 46]. *γ*‐Al_2_O_3_ exhibited the highest fraction of tetrahedral Al, consistent with its defective spinel‐like structure in which Al cations occupy both tetrahedral and octahedral sites. ZnAl_2_O_4_@*γ*‐Al_2_O_3_ showed a slightly reduced tetrahedral contribution due to the formation of a ZnAl_2_O_4_ shell at the surface of *γ*‐Al_2_O_3_ crystallites, where Zn^2+^ preferentially occupies tetrahedral sites. In contrast, ESA displayed a markedly broadened resonance in the octahedral region together with a significantly diminished tetrahedral signal, in line with the strong preference of Al for octahedral coordination in spinels [47]. The broad, asymmetric line shape reflects substantial local structural heterogeneity introduced by multication disorder, which produces a wide distribution of electric field gradients and pronounced quadrupolar broadening.

The Lewis acidity of alumina‐based materials originates primarily from coordinatively unsaturated surface Al sites, most notably tricoordinate and pentacoordinate environments [48]. Unfortunately, tricoordinate Al species are not directly detected due to their low abundance and fast T2 relaxation [45], and reliable quantification of pentacoordinate Al is complicated by overlap with the broadened octahedral resonance, particularly in ESA. Nonetheless, adsorption‐induced coordination changes can be inferred from variations in the tetrahedral and octahedral regions. After CF_4_ or H_2_O treatment, *γ*‐Al_2_O_3_ and ZnAl_2_O_4_@*γ*‐Al_2_O_3_ show increases in both regions, with more pronounced changes under H_2_O. Such behavior is consistent with strong H_2_O chemisorption and adsorption‐induced increases in Al coordination (tricoordinate → tetrahedral, pentacoordinate → octahedral). In contrast, ESA exhibits a stronger increase in the octahedral region after CF_4_ treatment, whereas the tetrahedral region increases more after H_2_O treatment. This indicates that pentacoordinate Al sites of ESA preferentially interact with CF_4_ over H_2_O. Given that pentacoordinate sites are generally more prevalent than tricoordinate sites [48], this can explain the improved resistance of ESA to H_2_O poisoning during CF_4_ hydrolysis.

In Al 2p x‐ray photoelectron spectroscopy (XPS) of the He‐treated samples, the binding energies of Al–O species [49] increased in the order *γ*‐Al_2_O_3_ (74.1 eV) < ZnAl_2_O_4_@*γ*‐Al_2_O_3_ (74.4 eV) < ESA (74.5 eV) (Figure 3d). In the O 1s spectra (Figure S13), the binding energy of the lattice oxygen [49] also increased in the order *γ*‐Al_2_O_3_ (531.1 eV) ≈ ZnAl_2_O_4_@*γ*‐Al_2_O_3_ (531.1 eV) < ESA (531.3 eV). The highest binding energies for ESA indicate that it contains Al and O atoms with the lowest electron densities. Upon CF_4_ exposure at 873 K, all samples exhibited slight positive shifts of the Al–O component in the Al 2p binding energy (Figure 3d). In addition, new shoulders appeared at higher binding energies (75.6–76.2 eV), which are attributable to the formation of surface Al–F species [50] generated during C–F activation. ESA exhibited the largest development of this shoulder, consistent with the fact that ESA can most efficiently activate C–F bonds. For completeness, XPS spectra of the other metal cations (Zn, Ga, Ni, Co) were also examined (Figure S14). High‐energy shoulders attributable to M–F bond formation (analogous to those of the Al–F features) were not observed for these metal elements. These results indicate that Al primarily acts as a binding site for F^–^, whereas the other metal cations do not stabilize F^–^ upon C–F activation. The F 1s spectra (Figure S15) showed a single fluoride (F^–^) peak for all samples [50], with binding energies decreasing in the order *γ*‐Al_2_O_3_ (685.6 eV) > ZnAl_2_O_4_@*γ*‐Al_2_O_3_ (685.3 eV) > ESA (685.1 eV). The largest downward shift of the F 1s peak for ESA, together with the upward shift of the Al–F component in the Al 2p region (Figure 3d), indicates the most strongly polarized Al–F bond in ESA.

The distinct adsorption behavior of CF_4_ and H_2_O on ESA arises from the electronic and structural consequences of its multication composition. In the entropy‐stabilized spinel lattice, heterometal coordination around O^2–^ generates a highly non‐uniform electrostatic field that effectively reduces oxygen basicity [51], as evidenced by the higher O 1s binding energy of lattice oxygen in ESA compared with *γ*‐Al_2_O_3_ and ZnAl_2_O_4_@*γ*‐Al_2_O_3_. The diminished oxygen basicity suppresses electron donation to neighboring Al centers, rendering Al more electron‐deficient, consistent with the highest Al 2p binding energy observed for ESA. This electronic modulation strengthens the hard Lewis‐acid character of Al, thereby enhancing stabilization of the hard F^–^ fragment formed during heterolytic C–F cleavage [52, 53, 54]. At the same time, reduced surface oxygen basicity disfavors proton transfer from H_2_O, weakening its dissociative adsorption and subsequent surface hydroxylation [55]. Taken together, these results demonstrate that the electron‐deficient Al–O motifs of ESA simultaneously enhance F^–^ stabilization while mitigating H_2_O poisoning, providing a unified mechanistic basis for its superior CF_4_ hydrolysis performance.

### 2.4 Reaction Mechanism for CF_4_ Hydrolysis

The mechanistic study of CF_4_ hydrolysis is experimentally challenging because the reaction occurs at high temperatures under strongly corrosive, fluorinating conditions. Nevertheless, earlier studies generally agree that the activation of the exceptionally stable first C–F bond constitutes the rate‐determining step in CF_4_ hydrolysis [42, 43]. This step is typically described as a heterolytic dissociation in which the CF_3_^δ+^ fragment is stabilized by surface Lewis basic O sites, whereas the F^δ–^ fragment binds to Lewis acidic Al centers. Consistent with this picture, diffuse reflectance infrared Fourier transform spectroscopy (DRIFTS) carried out after CF_4_ exposure on ESA and *γ*‐Al_2_O_3_ revealed a distinct *ν*(C–F) band characteristic of surface fluoroalkoxy‐like CF_x_‐O species [56] (Figure S16).

Conventionally, the high activity of Al‐containing oxides has been attributed to their abundance of Lewis acid sites. However, this interpretation is far from complete because many Al‐free transition‐metal oxides (e.g., TiO_2_, ZrO_2_, CeO_2_, Ga_2_O_3,_ ZnO, NiO, CoO) possess appreciable Lewis acidity (Table S3), yet they display negligible activity toward CF_4_ hydrolysis (Figure S17). We propose that the crucial distinction lies not in the quantity of Lewis acid sites but in the fundamentally different thermodynamic preferences of M–O versus M–F bond formation. For most transition‐metal cations, M–O bonds are substantially more stable than the corresponding M–F bonds, rendering these metals ineffective at stabilizing the F^–^ fragment in the presence of H_2_O. In contrast, Al^3+^ forms exceptionally strong Al–F bonds, with thermochemical data placing their bond strength in the range of ∼500–700 kJ mol^−1^ depending on the coordination environment [57]. This unusually high fluoride affinity makes the formation of surface Al–F species thermodynamically favored over Al–O bond formation, even under H_2_O‐rich reaction conditions. Consequently, the Al–O Lewis acid–base pair uniquely facilitates C–F bond activation by strongly stabilizing the F^–^ fragment, whereas other M–O species cannot provide such stabilization. Indeed, prior XPS results confirmed that F^–^ is primarily stabilized on Al sites rather than on other metal cations (Figure 3d and Figure S14).

**FIGURE 4:**
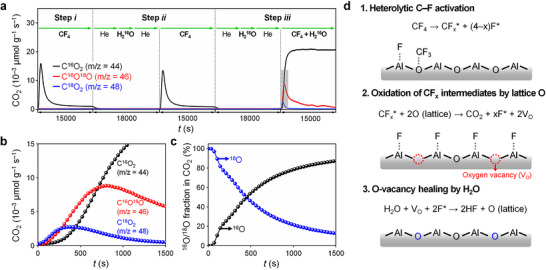
(a) The evolution of CO_2_ isotopologues during the sequential reaction experiment with the ESA catalyst at 873 K. Gas changes with time are summarized within the plot. (b) The CO_2_ isotopologue distribution immediately after introducing the CF_4_/H_2_^16^O mixture (magnified view of the shaded region in step iii of panel (a). (c) The corresponding ^16^O/^18^O distribution within the CO_2_ product. (d) Proposed MvK‐type reaction mechanism for CF_4_ hydrolysis over Al‐containing oxides.

Another key mechanistic question is whether lattice oxygen in Al‐containing oxides participates directly in CF_4_ hydrolysis. In the Mars–van Krevelen (MvK)‐type scenario, lattice oxygen is consumed during the oxidation of CF_x_ surface intermediates and subsequently replenished by H_2_O [42, 43]. An alternative possibility is a Langmuir–Hinshelwood‐type pathway in which surface‐chemisorbed CF_4_ and H_2_O react with one another [58]. To date, however, most mechanistic proposals have been based on theoretical calculations and lack direct experimental evidence [42, 43]. To address this question experimentally, we conducted a series of sequential reaction experiments over fully dehydrated ESA (Figure 4) and *γ*‐Al_2_O_3_ (Figure S18) and monitored the evolution of CO_2_ isotopologues by mass spectrometry. When dry CF_4_ was introduced over both samples (step i), a sharp C^16^O_2_ signal was detected even in the absence of H_2_O in the gas phase (Figure 4a and Figure S18a). This result demonstrates that lattice oxygen is consumed in the oxidation of CF_x_ surface intermediates and supports the involvement of an MvK‐type pathway. The C^16^O_2_ intensity gradually decreased with time, consistent with progressive depletion of lattice oxygen. Subsequent exposure to H_2_^16^O restored the C^16^O_2_ signal upon reintroduction of dry CF_4_ (step ii), indicating that H_2_O can replenish the oxygen vacancies.

To further track oxygen exchange, the catalysts were treated with H_2_^18^O vapor to incorporate ^18^O into the lattice, followed by reaction under a CF_4_/H_2_^16^O mixture (step iii). A preliminary experiment without catalysts confirmed that gas‐phase oxygen exchange between C^16^O_2_ and H_2_^18^O is negligible (Figure S19). The CO_2_ isotopologue distribution immediately after introducing the CF_4_/H_2_^16^O mixture is shown in Figure 4b and Figure S18b, and the corresponding ^16^O/^18^O distribution in the CO_2_ product is summarized in Figure 4c and Figure S18c. Notably, the formation of C^16^O_2_ is negligible during the initial reaction period (< 150 s), whereas ^18^O‐enriched CO_2_ (C^18^O_2_ and C^16^O^18^O) predominates. Thus, the CO_2_ produced at the onset of the reaction exclusively contains ^18^O originating from lattice oxygens. As lattice ^18^O is progressively consumed with increasing time‐on‐stream, the fraction of ^16^O‐containing CO_2_ increases. These results indicate that, despite supplying H_2_^16^O in the gas phase, initial CF_4_ hydrolysis primarily uses lattice ^18^O, providing compelling evidence for an MvK‐type mechanism. Both ESA (Figure 4) and *γ*‐Al_2_O_3_ (Figure S18) exhibit similar trends, indicating that the MvK mechanism is general to Al‐containing oxide catalysts. The only notable difference between them is the prolonged reaction of CF_4_ over *γ*‐Al_2_O_3_ in the absence of H_2_O (steps i and ii), which arises from the higher chemical lability of its lattice oxygen compared to that of ESA.

The aforementioned results indicate that CF_4_ hydrolysis over Al‐containing oxides proceeds through an MvK‐type pathway, as schematically illustrated in Figure 4d. The reaction is initiated by heterolytic C–F bond activation at Al–O Lewis acid‐base pairs, generating O‐bound CF_3_ (or more generally CF_x_) species together with Al‐bound F species. The subsequent oxidation of these CF_x_ intermediates is accomplished by lattice oxygen, and the consumed lattice oxygen is then replenished by dissociative H_2_O adsorption, thereby completing the catalytic cycle. Although these macroscopic features of the mechanism are experimentally well supported, the microscopic pathway by which surface CF_x_ species evolve to CO_2_ remains unresolved because the harsh, fluorinating conditions preclude direct spectroscopic detection of short‐lived surface intermediates. Existing density functional theory studies on *γ*‐Al_2_O_3_ and related surfaces generally predict stepwise C–F cleavage (CF_3_* → CF_2_* → CF*), followed by lattice‐oxygen insertion toward CO_2_ formation, but the precise sequence of transient oxidized CF_x_ species (e.g., CF_x_O) varies across computational models and has not converged to a single, widely accepted pathway [42].

### 2.5 Catalyst Stability During CF_4_ Hydrolysis

**FIGURE 5:**
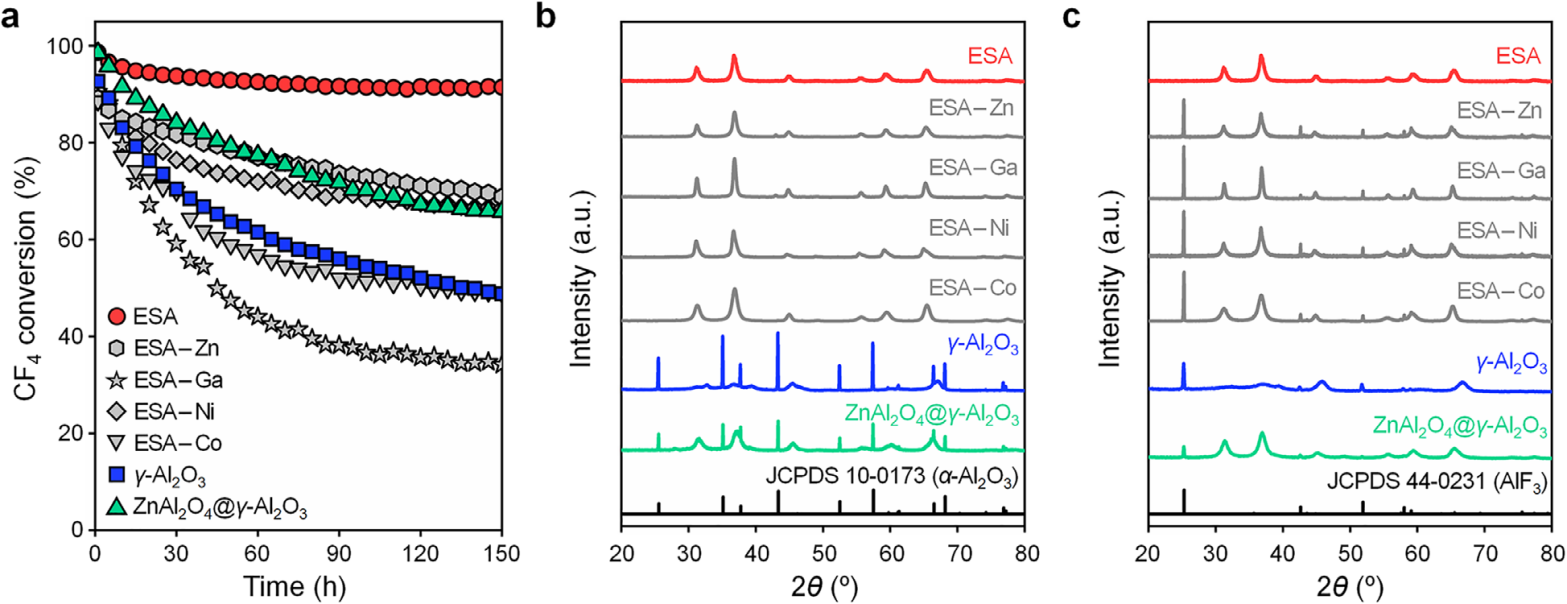
(a) Long‐term catalyst stability at 1073 K (reaction conditions: 0.67 kPa CF_4_, 0.67 kPa Ar, and 33 kPa H_2_O in He balance; CF_4_‐based WHSV = 0.2 h^−1^). (b) XRD patterns of the spent catalysts after 150 h of reaction. (c) XRD patterns of the catalysts treated under dry CF_4_ (reaction conditions: 1.0 kPa CF_4_ in He balance; CF_4_‐based WHSV = 0.2 h^−1^; 1073 K, 10 h).

The long‐term stability of all prepared catalysts (ESA, ESA–*elem*., *γ*‐Al_2_O_3_, and ZnAl_2_O_4_@*γ*‐Al_2_O_3_) was evaluated during CF_4_ hydrolysis at 1073 K (Figure 5a). The purpose of the stability test was to evaluate catalyst durability under more severe conditions (high temperature and high fluorine dose), rather than to operate at the optimal conditions. To impose accelerated deactivation conditions, the CF_4_‐based WHSV was increased 20‐fold relative to the earlier value (0.2 h^−1^ vs. 0.01 h^−1^) and the CF_4_ and H_2_O concentrations were also increased 2.7‐fold. This WHSV significantly exceeds previous literature values, providing a more stringent basis for evaluating catalyst stability (Table S4).

Conventional *γ*‐Al_2_O_3_ showed rapid deactivation over 150 h, with CF_4_ conversion decreasing from 92.7% to 48.8%. ZnAl_2_O_4_@*γ*‐Al_2_O_3_ exhibited improved stability but still experienced a gradual decline in CF_4_ conversion from 98.5% to 65.7%. The ESA—*elem*. samples generally exhibited poorer stability than ZnAl_2_O_4_@*γ*‐Al_2_O_3_. In stark contrast, ESA exhibited only a minor decrease in CF_4_ conversion from 98.6% to 91.6% during the first ∼100 h, after which the conversion remained essentially constant for the remainder of the test. These results clearly demonstrate that ESA exhibits not only enhanced activity but also superior stability under harsh CF_4_ hydrolysis conditions.

After 150 h of reaction, the crystal structure, surface area, and Lewis acid density of the spent catalysts were analyzed. According to XRD analysis (Figure 5b), the *γ*‐Al_2_O_3_ catalyst exhibited significant formation of the *α*‐Al_2_O_3_ phase, which has negligible Lewis acidity [59]. Due to the phase transformation and crystal ripening, the spent catalyst retained only 45% of the BET surface area and 40% of the Lewis acid density relative to the fresh material (Table 2). ZnAl_2_O_4_@*γ*‐Al_2_O_3_ exhibited reduced formation of *α*‐Al_2_O_3_ and greater retention of both BET surface area (55%) and Lewis acid density (50%). In contrast, no *α*‐Al_2_O_3_ formation was detected in the spent ESA and ESA–*elem*. catalysts (Figure 5b). The ESA–*elem*. catalysts exhibited more pronounced crystal ripening than ESA, resulting in lower retention of both BET surface area (51%–68%) and Lewis acid density (41%–62%), whereas ESA retained 77% of its BET surface area and 74% of its Lewis acid density (Table 2). The chemical states of heterometal cations in spent ESA also remained unchanged after the long‐term reaction, as confirmed by XPS (Figure S20). Consequently, the superior stability of ESA is attributed to its suppressed phase transition to *α*‐Al_2_O_3_ as well as its enhanced resistance to crystal ripening.

**TABLE 2:** Surface areas and Lewis acid densities of the catalysts before and after reaction.

Sample	*S*_BET_ ^a^ (m^2^ g^−1^)		*n*_Lewis_ ^b^ (µmol g^−1^)	
	Fresh	Spent^c^	Fresh	Spent^c^
ESA	101	78 (77%)	133	99 (74%)
ESA–Zn	89	61 (68%)	92	52 (57%)
ESA–Ga	68	39 (57%)	80	33 (41%)
ESA–Ni	74	48 (65%)	89	46 (62%)
ESA–Co	101	52 (51%)	107	49 (46%)
*γ*‐Al_2_O_3_	178	80 (45%)	210	85 (40%)
ZnAl_2_O_4_@*γ*‐Al_2_O_3_	143	79 (55%)	175	87 (50%)

^a^ BET surface areas calculated from N_2_ adsorption isotherms in the *P*/*P*_0_ range of 0.05–0.15.

^b^ Lewis acid densities determined by FT‐IR spectroscopy following pyridine adsorption at 423 K.

^c^ Spent catalysts collected after CF_4_ hydrolysis for 150 h (reaction conditions: 0.67 kPa CF_4_, 0.67 kPa Ar, and 33 kPa H_2_O in He balance; CF_4_‐based WHSV = 0.2 h^−1^).

The phase transition from *γ*‐Al_2_O_3_ to *α*‐Al_2_O_3_ typically occurs at temperatures above 1473 K [60], which is much higher than the present reaction temperature used for CF_4_ hydrolysis (1073 K). Thus, the formation of *α*‐Al_2_O_3_ under reaction conditions suggests the involvement of additional chemical pathways that substantially lower the transition barrier. We propose that this behavior arises from the formation of highly reactive aluminum fluoride species such as AlF_3_. AlF_3_ is commonly used as a mineralizer in the industrial synthesis of *α*‐Al_2_O_3_ because it significantly decreases the temperature required for its crystallization [61]. Partially hydrolyzed fluorides such as AlOF can also enhance Al cation mobility and readily transform into *α*‐Al_2_O_3_ in the presence of H_2_O [61, 62]. Under CF_4_ hydrolysis conditions where excess H_2_O is present, in situ formed AlF_3_ species are not directly detectable because they rapidly hydrolyze to *α*‐Al_2_O_3_. Thus, to probe in situ AlF_3_ formation more directly, the catalysts were treated with dry CF_4_ (Figure 5c). After dry CF_4_ treatment, *γ*‐Al_2_O_3_ indeed exhibited intense XRD peaks corresponding to crystalline AlF_3_, whereas ZnAl_2_O_4_@*γ*‐Al_2_O_3_ showed diminished but still measurable formation of AlF_3_. In stark contrast, ESA exhibited no detectable formation of AlF_3_. Therefore, the enhanced stability of ESA can be attributed to its superior resistance to the in situ formation of AlF_3_ under the fluorinating reaction conditions. Interestingly, the ESA–*elem*. catalysts exhibited significant AlF_3_ formation but showed no detectable formation of *α*‐Al_2_O_3_ after reaction. This observation suggests that multication incorporation thermodynamically inhibits the formation of the *α*‐Al_2_O_3_ phase, even after the formation of AlF_3_. However, the in situ formation of AlF_3_ might still enhance Al cation mobility, leading to accelerated crystal ripening.

At first glance, the high stability of ESA may appear contradictory, since earlier results showed that ESA efficiently activates CF_4_ and forms Al–F species during catalysis. This raises a natural question: if ESA forms Al–F bonds so efficiently, why is crystalline AlF_3_ formed on *γ*‐Al_2_O_3_ but not on ESA under fluorinating conditions? This apparent contradiction is resolved by recognizing the fundamental distinction between surface fluorination, which is essential for C–F bond activation, and bulk fluorination, which leads to lattice destabilization and AlF_3_ formation. ESA efficiently forms surface Al–F species that enable heterolytic C–F cleavage; however, its high configurational entropy inhibits the propagation of fluorination into the bulk lattice and thereby prevents the nucleation and growth of crystalline AlF_3_.

## 3 Conclusion

The present results demonstrate that ESA breaks the conventional activity–stability tradeoff in CF_4_ hydrolysis by combining a multication spinel framework with an entropy‐stabilized lattice. The incorporation of multiple heterometal cations produces an electron‐deficient Al–O environment that enhances heterolytic C–F bond activation while simultaneously suppressing H_2_O poisoning, thereby enabling high intrinsic activity. The catalyst exhibits high activity not only in the hydrolysis of CF_4_ but also C_2_F_6_ (Figure S21), indicating its potential applicability to other perfluorocarbons. At the same time, the high configurational entropy of ESA inhibits the propagation of fluorination into the bulk lattice, effectively suppressing in situ AlF_3_ formation and the subsequent hydrolysis into catalytically inactive *α*‐Al_2_O_3_. Beyond these structure–property relationships, isotope‐labeling experiments provide direct evidence that CF_4_ hydrolysis on Al‐based oxides proceeds through a Mars–van Krevelen‐type mechanism, in which lattice oxygen oxidizes CF_x_ intermediates and the resulting oxygen vacancies are replenished by H_2_O. Notably, ESA should be regarded not as a single optimized formulation but as a promising materials platform. The broad compositional space of entropy‐stabilized aluminates enables tuning of surface electronic structure, Lewis acidity, and fluorination resistance, offering a promising pathway toward next‐generation catalysts for CF_4_ and related perfluorinated gases.

## Author Contributions

**Seunghyuck Chi**: validation, investigation, data curation, writing – review and editing, writing – original draft, visualization. **Hyungmin Jeon**: data curation, investigation, visualization, formal analysis, writing – review and editing. **Yaejun Baik**: formal analysis, data curation, investigation. **DongHwan Oh**: data curation, formal analysis, investigation. **Jin Seok**: investigation, formal analysis, data curation. **Woosung Choi**: formal analysis. **Seungjun Lee**: formal analysis. **Minkee Choi**: conceptualization, funding acquisition, project administration, resources, supervision, writing – review and editing, writing – original draft.

## Conflicts of Interest

The authors declare no conflicts of interest.

## Supporting information




**Supporting File**: anie73019‐sup‐0001‐SuppMat.docx.

## Data Availability

The data that support the findings of this study are available from the corresponding author upon reasonable request.
