# Supplementary material for: Entropy‐Stabilized Aluminate Catalysts That Break the Activity–Stability Tradeoff in CF4 Hydrolysis
Source: Angew Chem Int Ed Engl. 2026 Jun 8;65(32):e6752036. doi: 10.1002/anie.6752036 (PMC13427189; doi:10.1002/anie.6752036)
Supplement: Supplementary file 1 — Supporting File: anie73019‐sup‐0001‐SuppMat.docx. [file ANIE-65-e6752036-s001.docx]

**Supporting Information**

**Entropy-Stabilized Aluminate Catalysts that Break the Activity–Stability Tradeoff in CF_4_ Hydrolysis**

Seunghyuck Chi,^1^ Hyungmin Jeon,^1^ Yaejun Baik,^1^ DongHwan Oh,^1^ Jin Seok,^1^ Woosung Choi,^2^ Seungjun Lee,^2^ and Minkee Choi^1,*^

^1^*Department of Chemical and Biomolecular Engineering (BK21 Four), Korea Advanced Institute of Science and Technology (KAIST), Daejeon 34141, Republic of Korea*

^2^*EHS/Infra Technology Research Center, Samsung Electronics Co., Ltd., Hwaseong 18448, Republic of Korea*

*Corresponding author. E-mail: [mkchoi@kaist.ac.kr](mailto:mkchoi@kaist.ac.kr) (M. Choi)

**Experimental section**

**Catalyst Preparation.** ESA containing Al, Zn, Ga, Ni, and Co was synthesized using pseudoboehmite (Pural SB, Sasol) as the Al source and metal nitrate salts as non-Al precursors. In a typical procedure, 2.7 g of Zn(NO_3_)_2_·6H_2_O (98%, Sigma-Aldrich), 3.8 g of Ga(NO_3_)_3_·9H_2_O (99%, Sigma-Aldrich), 2.6 g of Ni(NO_3_)_2_·6H_2_O (99%, Sigma-Aldrich), and 2.6 g of Co(NO_3_)_2_·6H_2_O (98%, Sigma-Aldrich) were dissolved in 100 g of deionized water, followed by the addition of 5.0 g of pseudoboehmite (molar ratio Al : Zn : Ga : Ni : Co = 8 : 1 : 1 : 1 : 1). The mixture was vigorously stirred at room temperature for 3 h, transferred to a Teflon-lined stainless-steel autoclave, and hydrothermally treated at 443 K with tumbling (60 rpm) for 24 h. The resulting solid was dried at 373 K and calcined in dry air at 1073 K (heating rate: 2 K min^–1^) for 6 h. For comparison, ESA – *elem.* samples were prepared in the same manner, each omitting one of the constituent elements (Zn, Ga, Ni, or Co) from the ESA formulation. *γ*-Al_2_O_3_ and ZnAl_2_O_4_@*γ*-Al_2_O_3_ (Zn/Al molar ratio = 0.1) were synthesized by adding 5.0 g of pseudoboehmite to aqueous solutions containing 1.6 g of HNO_3_ (60%, Samchun) or 2.2 g of Zn(NO_3_)_2_·6H_2_O (98%, Sigma-Aldrich), respectively, in 100 g of deionized water. The mixtures were subjected to the same hydrothermal treatment, drying, and calcination procedures as described above. Commercial TiO_2_ (anatase, 99.7%, Sigma-Aldrich), ZrO_2_ (99%, Sigma-Aldrich), CeO_2_ (99.5%, Daejung), Ga_2_O_3_ (99.9%, Sigma-Aldrich), ZnO (99.9%, Sigma-Aldrich), NiO (99%, Sigma-Aldrich), and CoO (99%, Sigma-Aldrich) were purchased and used as received without further treatment.

**Characterization.** X-ray diffraction (XRD) patterns were collected on a SmartLab diffractometer (Rigaku) equipped with Cu Kα radiation (45 kV, 200 mA). For in situ high-temperature XRD, as-synthesized samples obtained after hydrothermal treatment were heated under flowing air (50 cm^3^ min^–1^) to each target temperature (773, 873, 973, and 1073 K (heating rate: 5 K min^–1^), and XRD patterns were collected after holding for 1 h. A graphite monochromator was used to eliminate fluorescence arising from cobalt-containing samples. Transmission electron microscopy (TEM), high-angle annular dark-field scanning transmission electron microscopy (HAADF-STEM), and energy dispersive spectroscopy (EDS) elemental mapping were performed on a Titan Cubed G2 60–300 microscope operated at 300 kV. Samples were dispersed in ethanol, deposited onto a lacey carbon film supported on a 300-mesh Au grid, and dried prior to imaging. Bulk elemental compositions were determined by inductively coupled plasma optical emission spectrometry (ICP-OES, Thermo Scientific iCAP 6300). Temperature-programmed reduction (H_2_-TPR) was carried out using a BELCAT II instrument (BEL Japan) equipped with a thermal conductivity detector. Typically, 0.5 g of sieved catalyst particles (200–300 μm) were pretreated under flowing Ar (50 cm^3^ min^–1^) at 973 K for 2 h and then cooled to 373 K. The temperature was subsequently ramped from 373 to 1073 K at 10 K min^–1^ under a flowing 3.9% H_2_/Ar mixture (50 cm^3^ min^–1^). N_2_ adsorption–desorption isotherms were measured at 77 K using a BELSORP-MAX volumetric analyzer (BEL Japan) after vacuum degassing at 673 K for 4 h. Brunauer–Emmett–Teller (BET) surface areas were calculated in the *P*/*P*_0_ range of 0.05–0.15, and total pore volumes were obtained at *P*/*P*_0_ = 0.95. Pore size distributions were determined by nonlocal density functional theory (NLDFT) analysis, assuming a metal oxide with cylindrical pore geometry.

Fourier transform infrared (FT-IR) spectroscopy following pyridine adsorption was conducted on a Nicolet iS50 spectrometer (Thermo Scientific). A self-supporting wafer containing 30 mg of sample was placed in an in situ FT-IR cell equipped with CaF_2_ windows. The sample was pretreated under vacuum at 673 K for 4 h. After cooling to 423 K, background spectra were collected. Pyridine adsorption was carried out by introducing pyridine vapor saturated at 298 K into the cell at 423 K for 1 h, followed by evacuation for 2 h at the same temperature to remove weakly adsorbed species. Spectra were recorded at a resolution of 4.0 cm^–1^. The density of Lewis acid sites was quantified from the integrated intensity of the band at 1455 cm^–1^ using an extinction coefficient of ε = 2.22 cm μmol^–1^. Diffuse reflectance infrared Fourier transform spectroscopy (DRIFTS) after CF_4_ adsorption was also conducted on the same instrument, using a PIKE diffuse IR cell accessory. Prior to CF_4_ adsorption, the samples were pretreated under flowing He at 773 K for 2 h, and cooled to 373 K, at which background spectra were collected. Then, the sample was treated under flowing CF_4_ at 773 K for 1 h, and cooled to 373 K, followed by He purging at the same temperature for 1 h to remove weakly adsorbed CF_4_. DRIFTS spectra were recorded with a resolution of 4.0 cm^–1^ by averaging 64 scans.

Temperature-programmed desorption mass spectrometry (TPD–MS) was carried out in a fixed-bed quartz reactor (inner diameter = 10.9 mm) coupled to an online quadrupole mass spectrometer (OmniStar GSD 320, Pfeiffer Vacuum). Typically, 0.3 g of sieved catalyst particles (200–300 μm) were loaded and degassed in He (50 cm^3^ min^–1^) at 873 K for 2 h. For CF_4_ desorption experiments, 1% CF_4_/He (50 cm^3^ min^–1^) was introduced at 873 K for 1 h, after which the sample was cooled to 373 K while maintaining the CF_4_/He flow. The gas was then switched to He (50 cm^3^ min^–1^), and the sample was purged for 1 h at 373 K to remove weakly adsorbed species. CF_4_ desorption profiles were collected by ramping the temperature from 373 to 1073 K at 10 K min^–1^ under He flow (50 cm^3^ min^–1^). H_2_O desorption experiments were conducted using a similar protocol, except that a 33% H_2_O/He mixture (50 cm^3^ min^–1^) was used in place of 1% CF_4_/He during the adsorption and cooling steps.

X-ray photoelectron spectroscopy (XPS) was performed using an Axis Supra spectrometer (Kratos) equipped with a monochromatic Al Kα radiation source (1486.7 eV). All samples were pretreated in He or CF_4_ (1%, He balance) at 873 K for 4 h and then placed in a sample holder inside an argon-filled glovebox (O_2_ < 1.0 ppm, H_2_O < 1.0 ppm). The sample holder was transferred directly from the glovebox to the connected analysis chamber without air exposure. Binding energies were calibrated to the C 1s peak at 284.5 eV. To minimize fitting bias, all XPS peak deconvolutions were performed using identical full widths at half maximum (FWHM) values for corresponding components. Solid-state ^27^Al magic-angle spinning nuclear magnetic resonance (^27^Al MAS NMR) spectra were collected on a Bruker Avance III HD spectrometer (Bruker) equipped with a 9.4 T magnet and a two-channel DVT probe. Samples were pretreated in flowing He, CF_4_ (1%, He balance), or H_2_O vapor (33%, He balance) at 873 K for 4 h, and transferred into 4 mm MAS rotors inside an argon-filled glovebox using a 4 mm MAS rotor disposable insert kit (Bruker BioSpin, B4493) to prevent air exposure. Spectra were recorded using a one-pulse sequence with a MAS rate of 12 kHz, a ^27^Al resonance frequency of 104.3 MHz, a pulse width of 4 µs, a relaxation delay of 0.5 s, a spectral width of 750 ppm, an offset of 0 ppm, and 1024 scans.

**Catalytic Measurements.** Catalytic hydrolysis of CF_4_ was conducted under atmospheric pressure in a tubular Inconel reactor (inner diameter = 10.9 mm) connected to a Teflon-lined water trap and a silica gel trap (bead size: 3.5 mm, Sigma-Aldrich) for the removal of H_2_O and HF, respectively. Reaction effluents were analyzed using an online gas chromatograph (GC, Agilent) equipped with a Porapak Q column (Supelco, 6 ft × 1/8 in × 2.1 mm) and a thermal conductivity detector. Typically, 2.0 g of catalysts (pressed and sieved to 200–300 μm) were loaded into the reactor and pretreated in flowing He at 773 K for 2 h. After pretreatment, temperature-dependent catalytic measurements were carried out by heating the reactor at a rate of 2 K min^–1^. At every 20 K increment, the temperature was held for 1 h, and catalytic performance was evaluated under a reactant feed composed of 0.25 kPa CF_4_, 0.25 kPa Ar as an internal standard, and 12.7 kPa H_2_O vapor balanced with He, corresponding to a CF_4_-based weight hourly space velocity (WHSV) of 0.01 h^–1^. For all kinetic measurements, CF_4_ conversion was kept low (< 20%) to ensure kinetic relevance. Apparent activation energies were obtained from Arrhenius plots over the temperature range of 803–843 K. Reaction orders with respect to CF_4_ and H_2_O were evaluated at 823 K by varying the partial pressures of CF_4_ (0.27–0.67 kPa) and H_2_O (2.8–33 kPa) with a He balance. Long-term stability tests were conducted at 1073 K under atmospheric pressure. The reaction was performed under a feed composed of 0.67 kPa CF_4_, 0.67 kPa Ar, and 33 kPa H_2_O balanced with He, corresponding to a CF_4_-based WHSV of 0.2 h^–1^ to impose accelerated deactivation conditions.

**Isotope-Labeling Experiments.** To examine the participation of lattice oxygen in CF_4_ hydrolysis, oxygen-isotope labeling experiments were conducted under atmospheric pressure in a tubular Inconel reactor (inner diameter = 10.9 mm). Typically, 0.5 g of sieved catalyst particles (200–300 μm) were pretreated in He at 873 K. For the isotope-labeling sequence, the catalyst was subsequently exposed to (1) 1.0 kPa CF_4_/He, (2) 33 kPa H_2_^16^O/He, (3) 1.0 kPa CF_4_/He, (4) 33 kPa H_2_^18^O/He, and (5) 0.67 kPa CF_4_ and 33 kPa H_2_^16^O in He, with a total gas flow rate of 30 cm^3^ min^–1^. Between each step, the reactor was purged with He (30 cm^3^ min^–1^) for 2 h. After passing through a Teflon-lined water trap and a silica gel trap to remove H_2_O and HF, the product stream was analyzed using an online quadrupole mass spectrometer (OmniStar GSD 320, Pfeiffer Vacuum). A catalyst-free blank experiment was conducted in the same reactor packed only with quartz wool at 873 K under a flow of 33.3 kPa H_2_^18^O balanced with C^16^O_2_ with a total gas flow rate of 30 cm^3^ min^–1^.


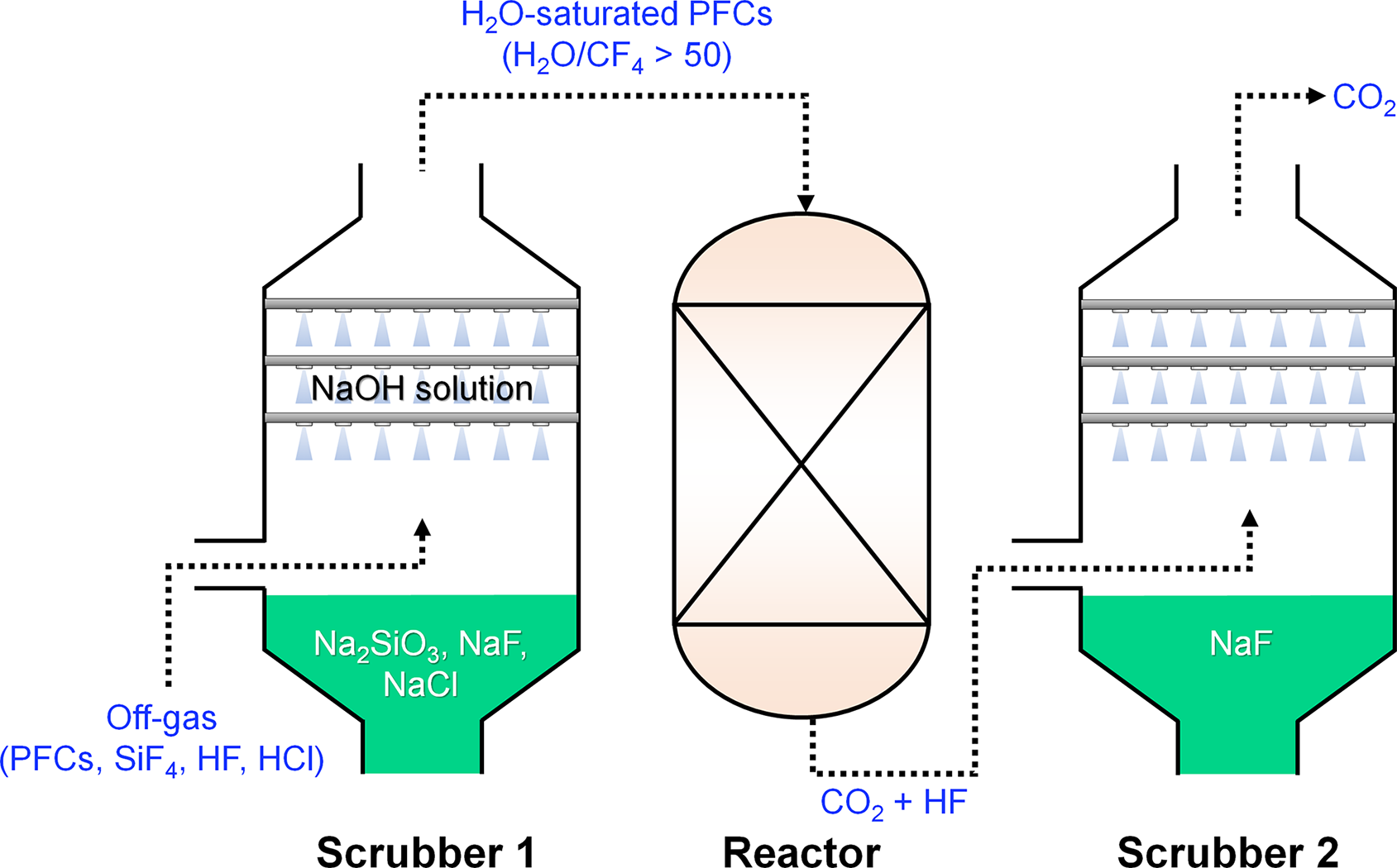


**Figure S1.** Schematic diagram of perfluorocarbon (PFC) hydrolysis in semiconductor manufacturing processes. During semiconductor manufacturing, various byproducts, including SiF_4_, HF, and HCl, are generated in addition to unreacted PFCs. The off-gases are first scrubbed with an aqueous NaOH solution to remove species other than PFCs. Subsequently, the resulting H_2_O-saturated PFC stream is introduced into a catalytic converter for hydrolysis. After catalytic hydrolysis, the generated HF is removed again through a downstream scrubbing process.


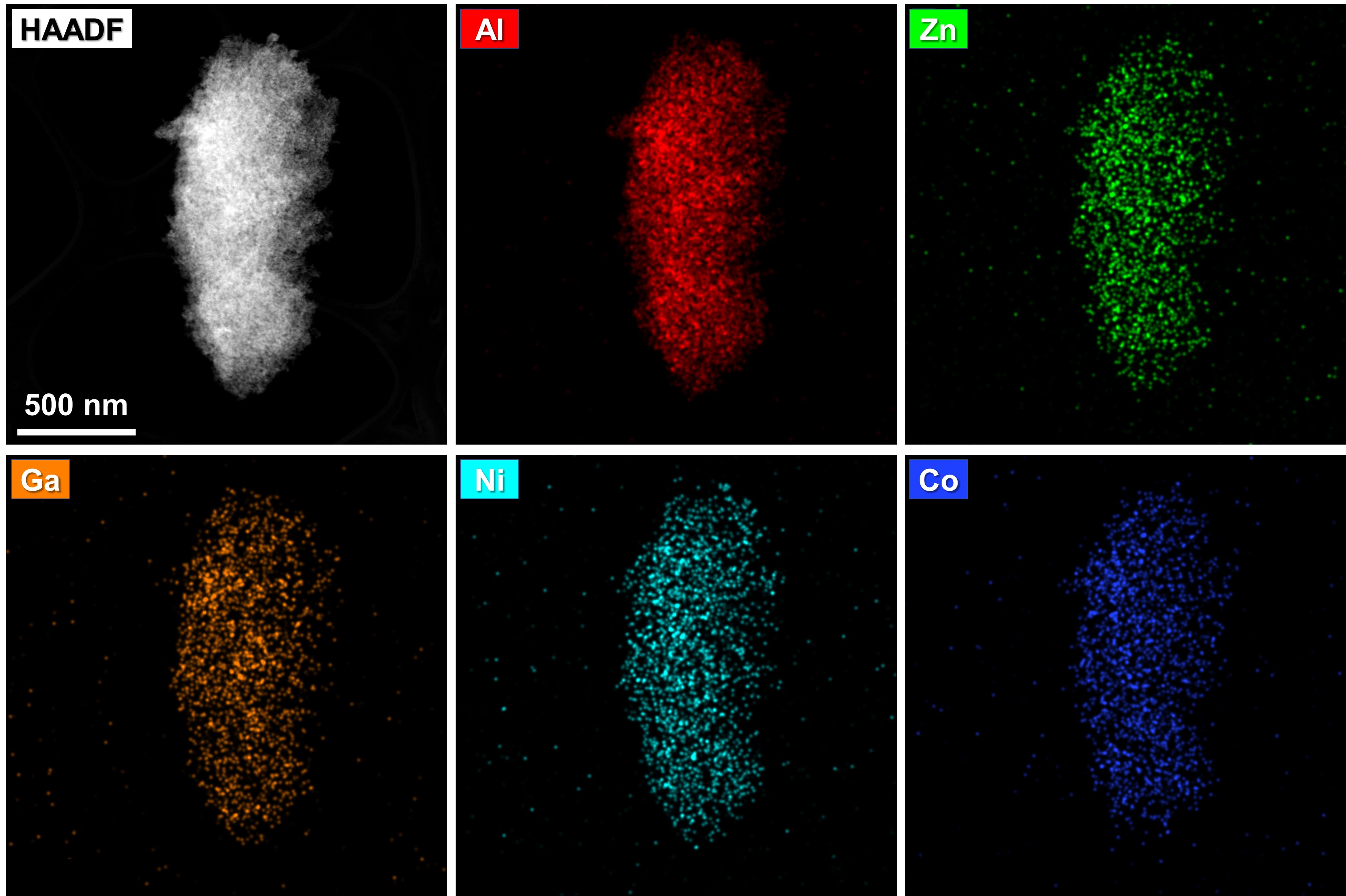


**Figure S2.** Low-magnification EDS mapping image of ESA.

**
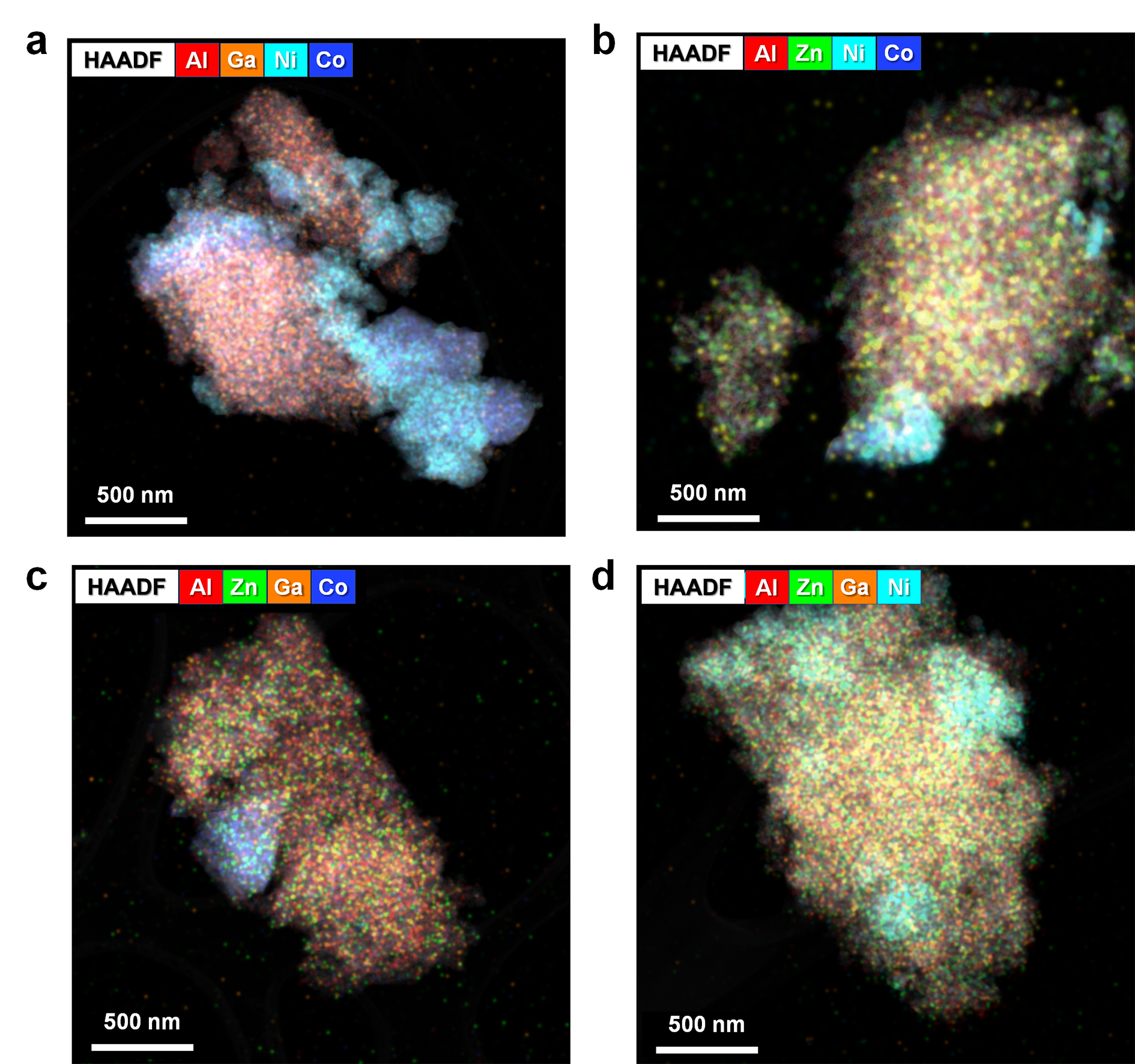
**

**Figure S3.** Low-magnification EDS mapping images of (a) ESA – Zn, (b) ESA – Ga, (c) ESA – Ni, and (d) ESA – Co.

**
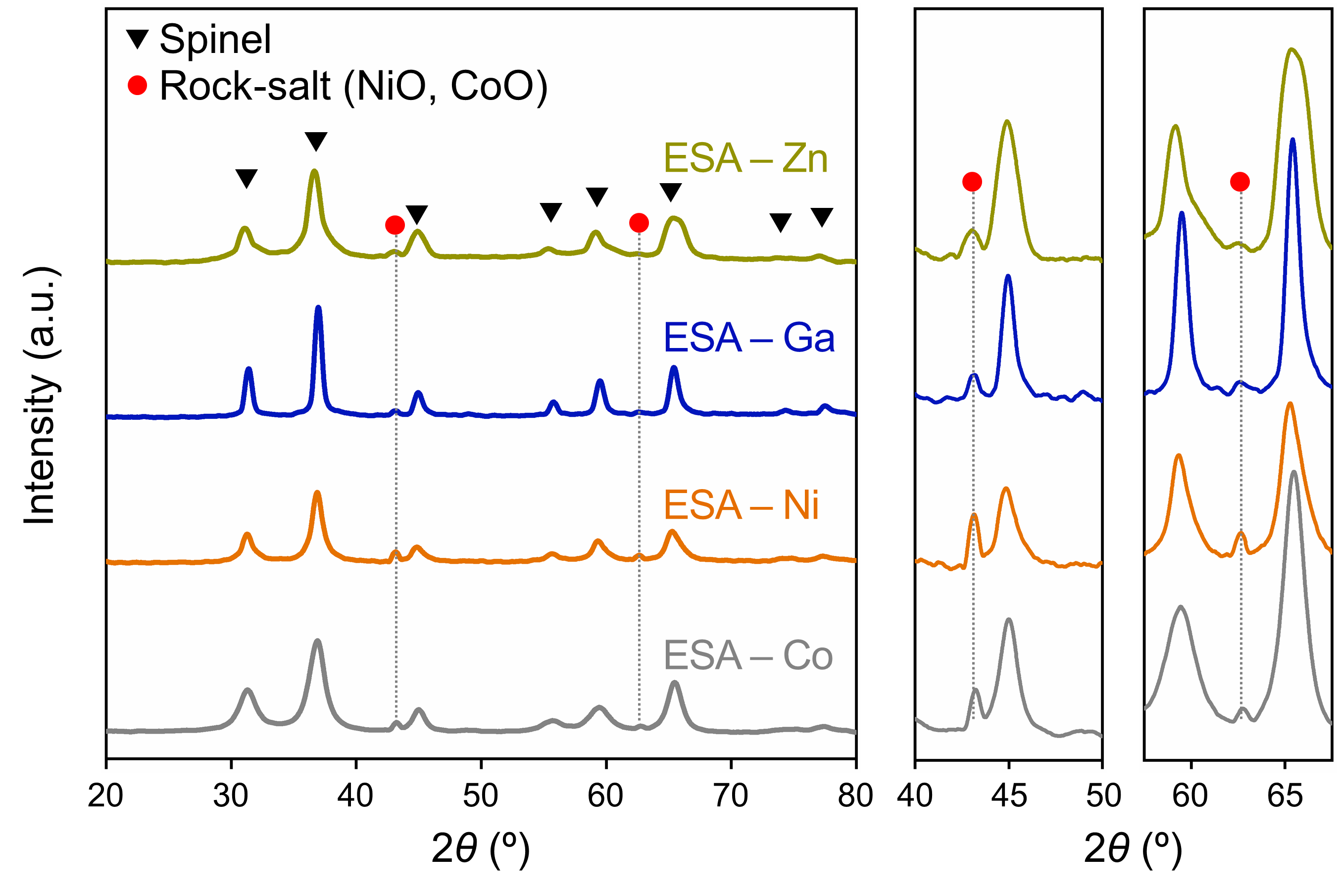
**

**Figure S4.** XRD patterns of ESA – *elem.* samples. Enlarged views of the selected 2*θ* regions are provided on the right to highlight the rock-salt reflections.


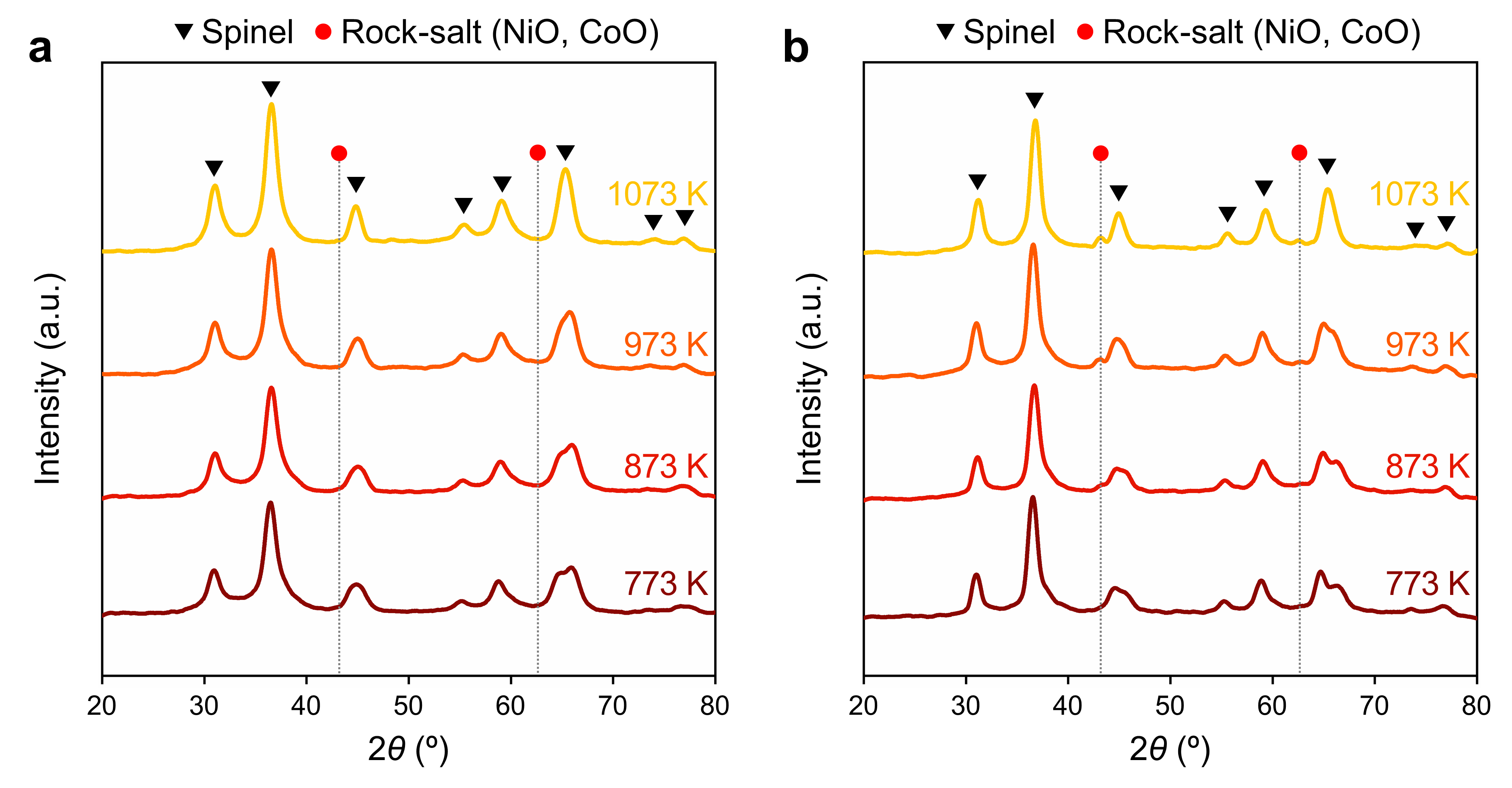


**Figure S5.** In situ high-temperature XRD patterns of (a) ESA and (b) ESA – Ga in the temperature range of 773–1073 K.

**
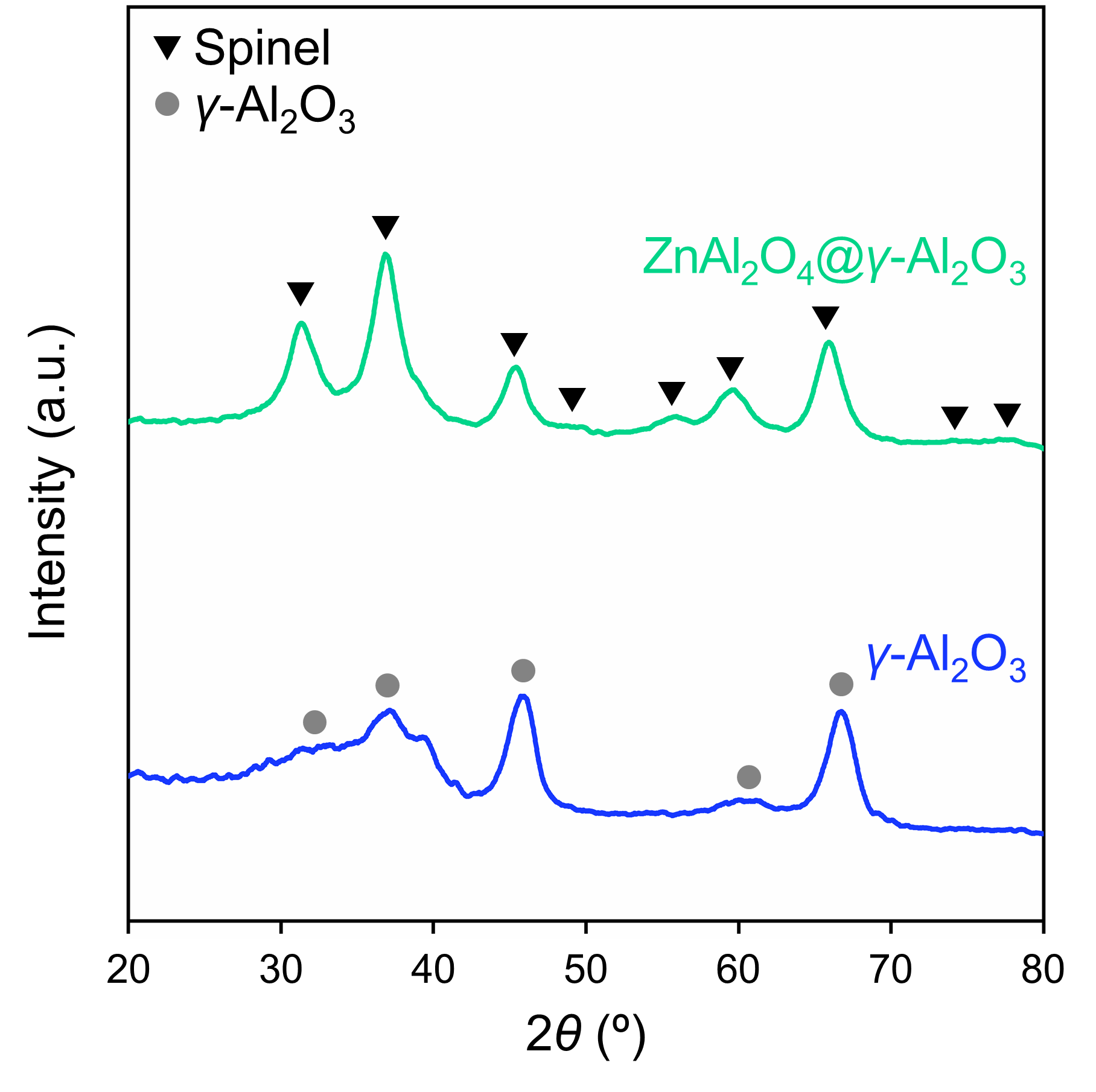
**

**Figure S6.** XRD patterns of *γ*-Al_2_O_3_ and ZnAl_2_O_4_@*γ*-Al_2_O_3_.


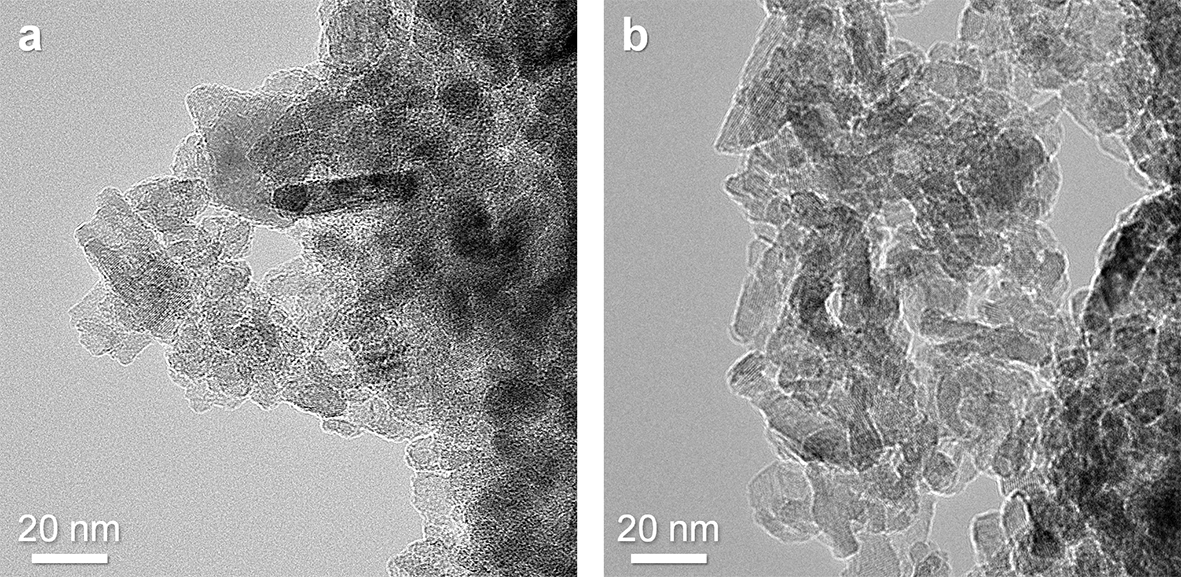


**Figure S7.** TEM images of (a) *γ*-Al_2_O_3_ and (b) ZnAl_2_O_4_@*γ*-Al_2_O_3_.

**
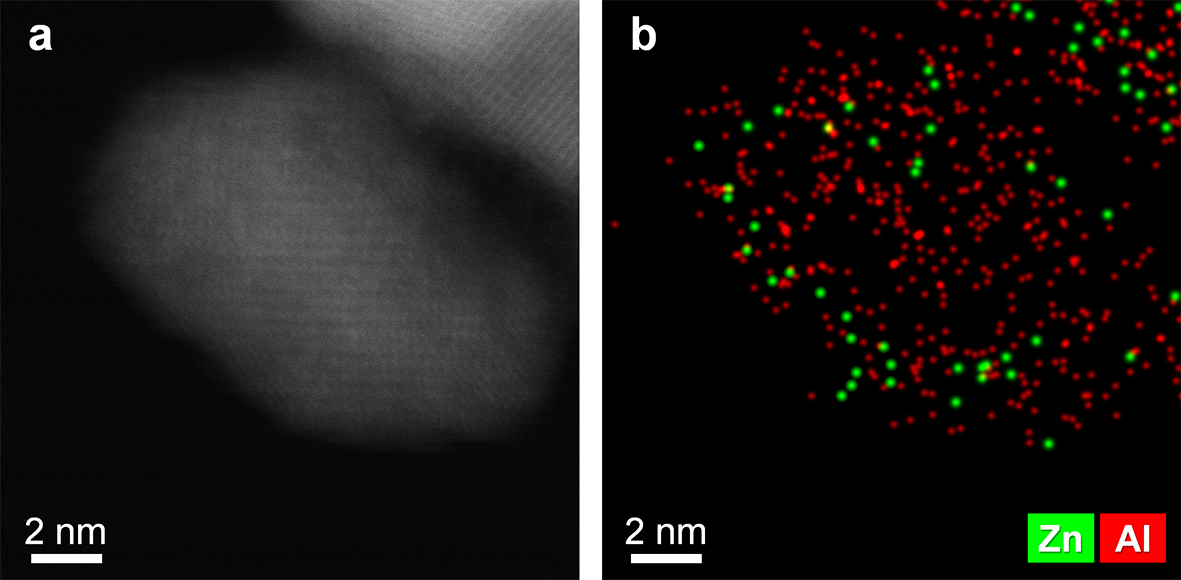
**

**Figure S8.** (a) HAADF-STEM and (b) EDS mapping images of ZnAl_2_O_4_@*γ*-Al_2_O_3_.

**
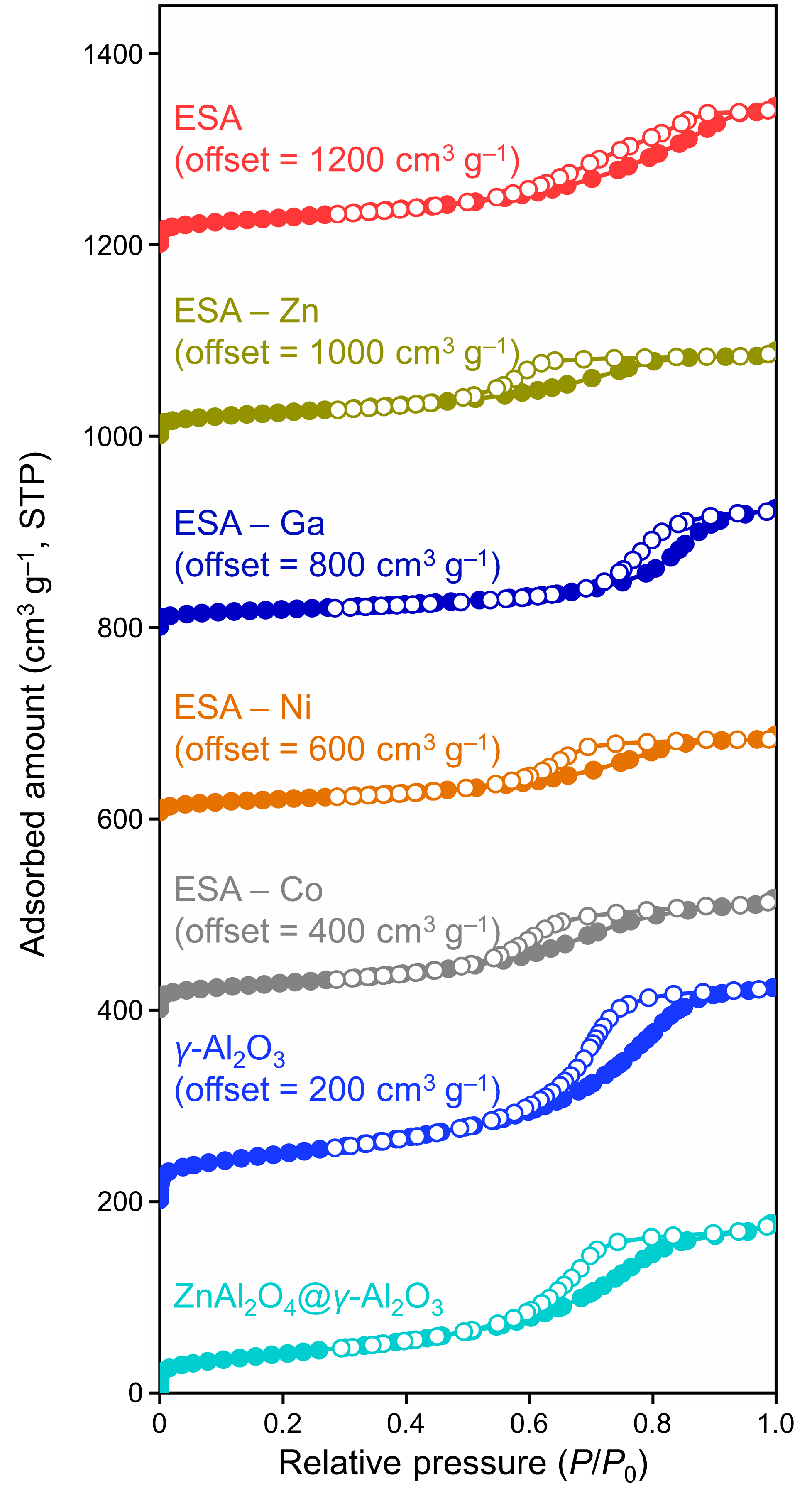
**

**Figure S9.** N_2_ adsorption–desorption isotherms of all catalyst samples at 77 K.

**
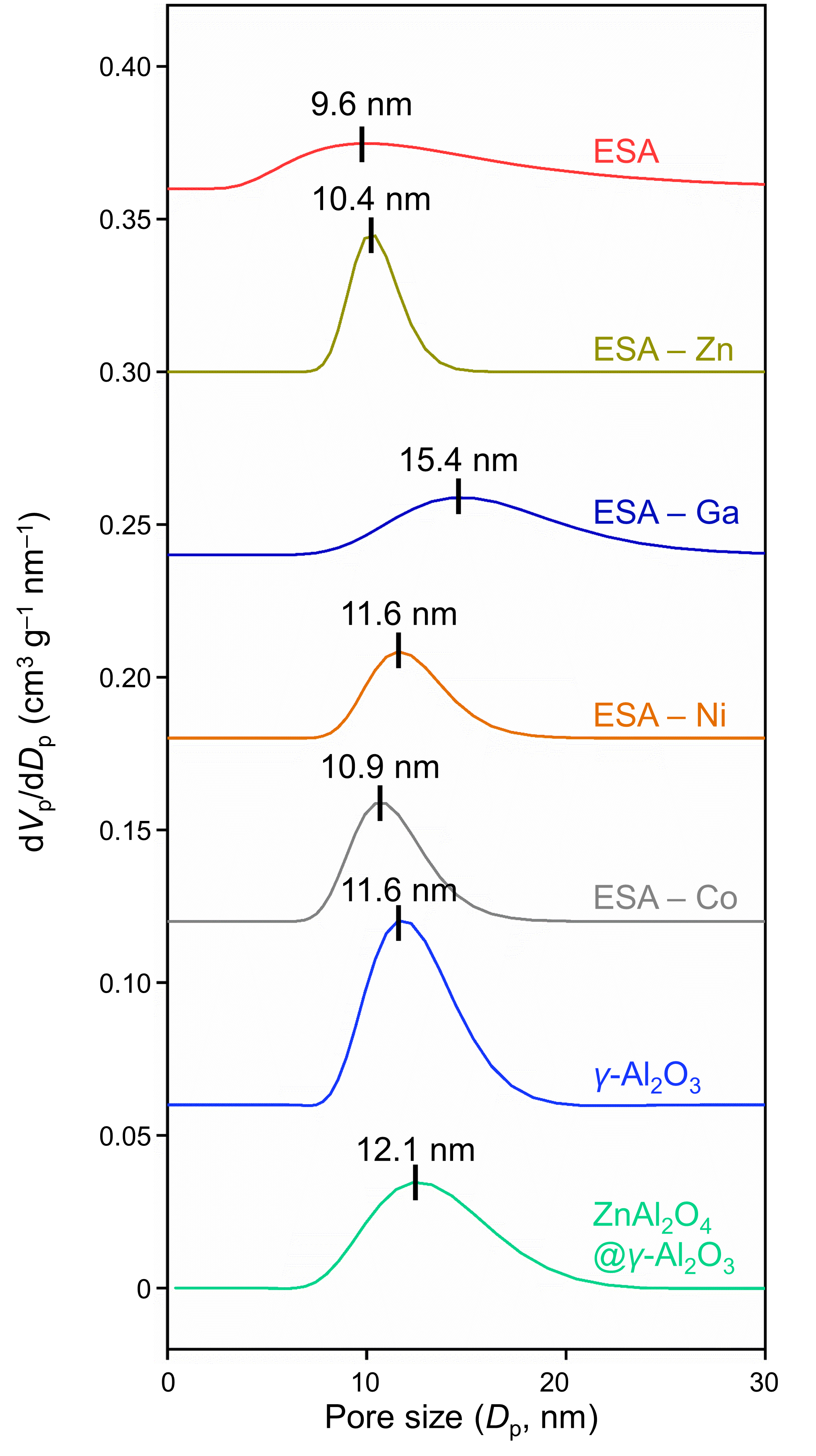
**

**Figure S10.** Pore size distributions of the catalysts determined by NLDFT calculations.

**
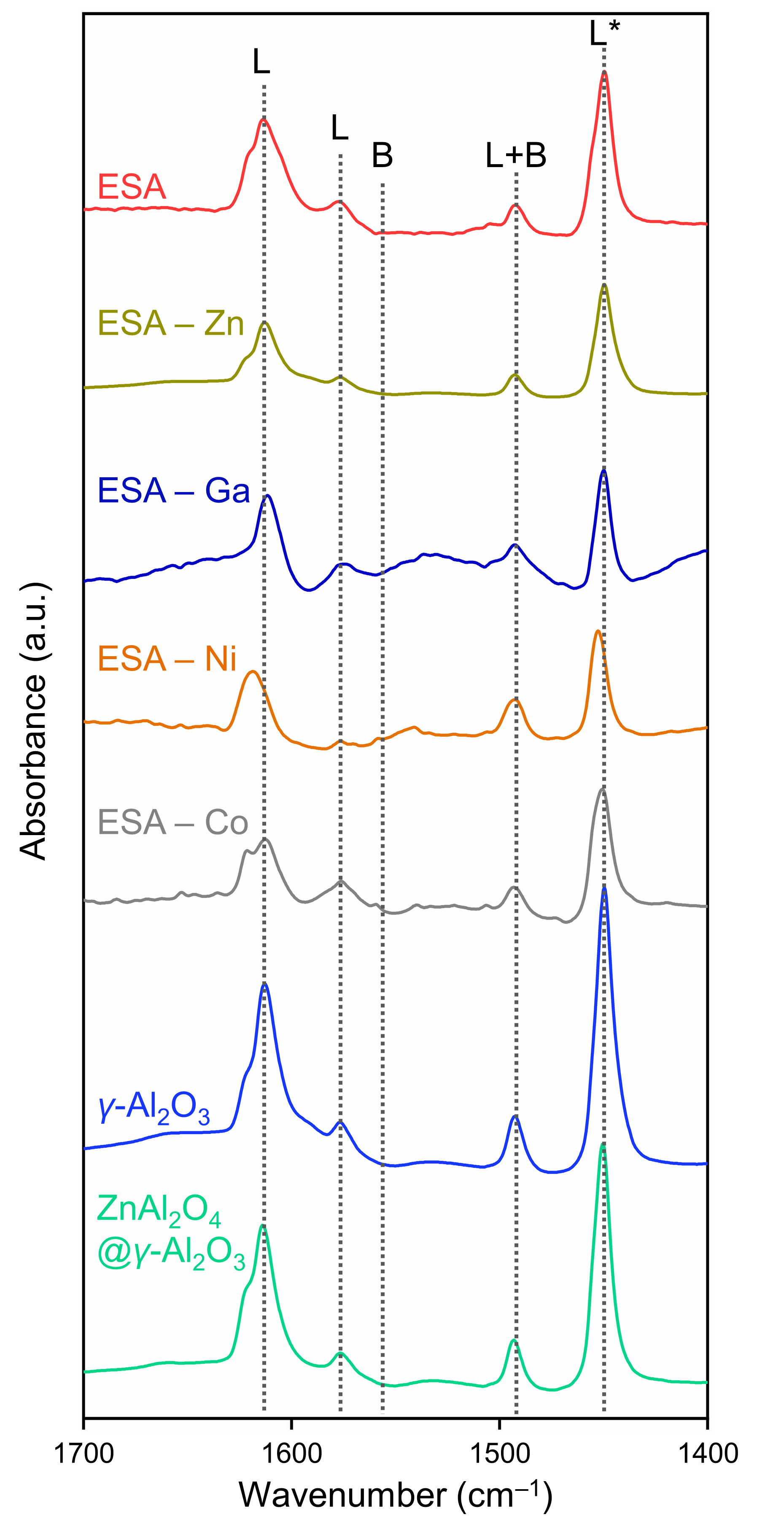
**

**Figure S11.** FT-IR spectra of the catalysts after pyridine adsorption at 423 K (L: Lewis acid sites, B: Brønsted acid sites). The asterisk denotes the band used for quantification.

**
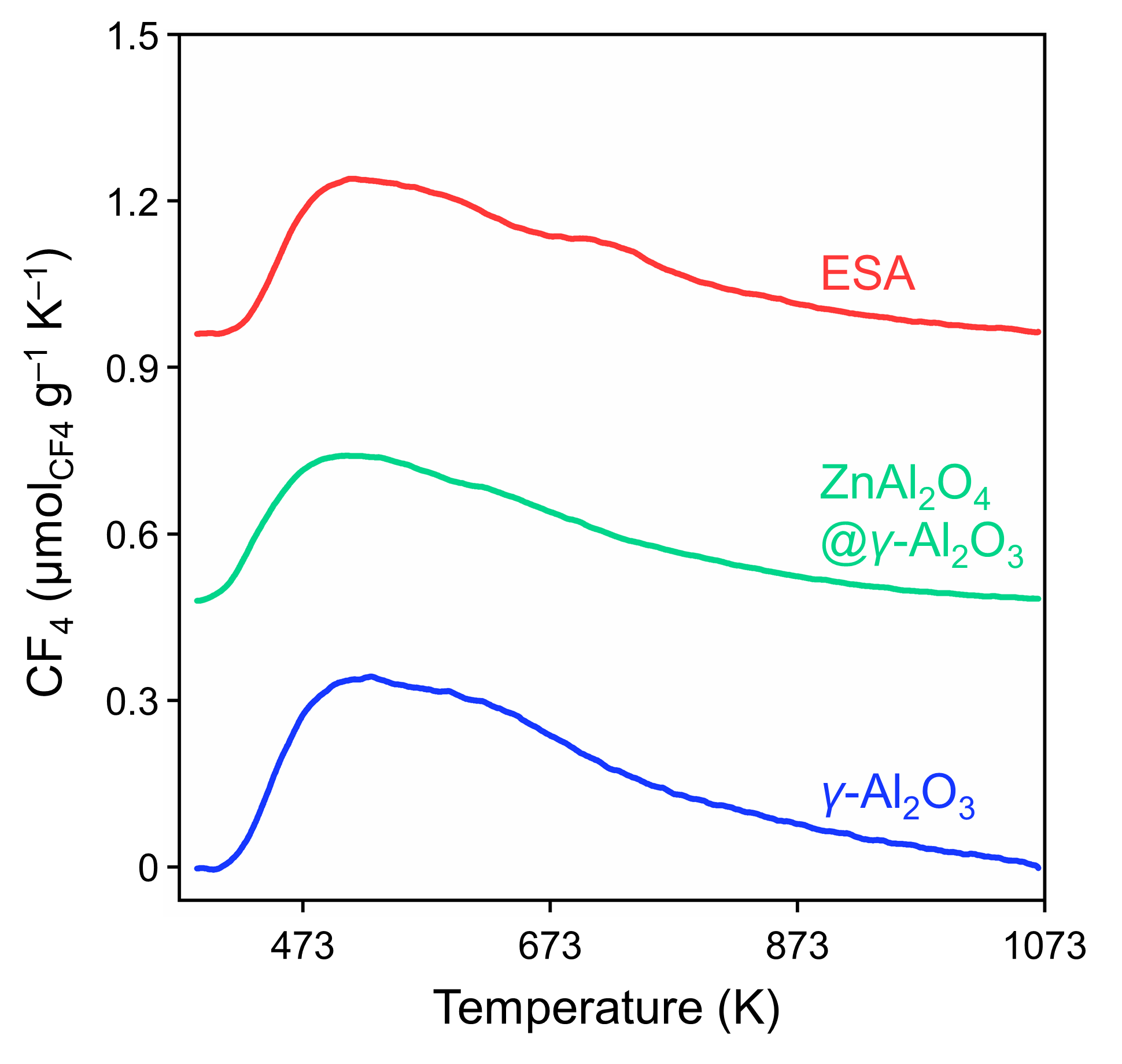
**

**Figure S12.** CF_4_ TPD–MS profiles of the catalysts after CF_4_ pretreatment at 373 K.

**
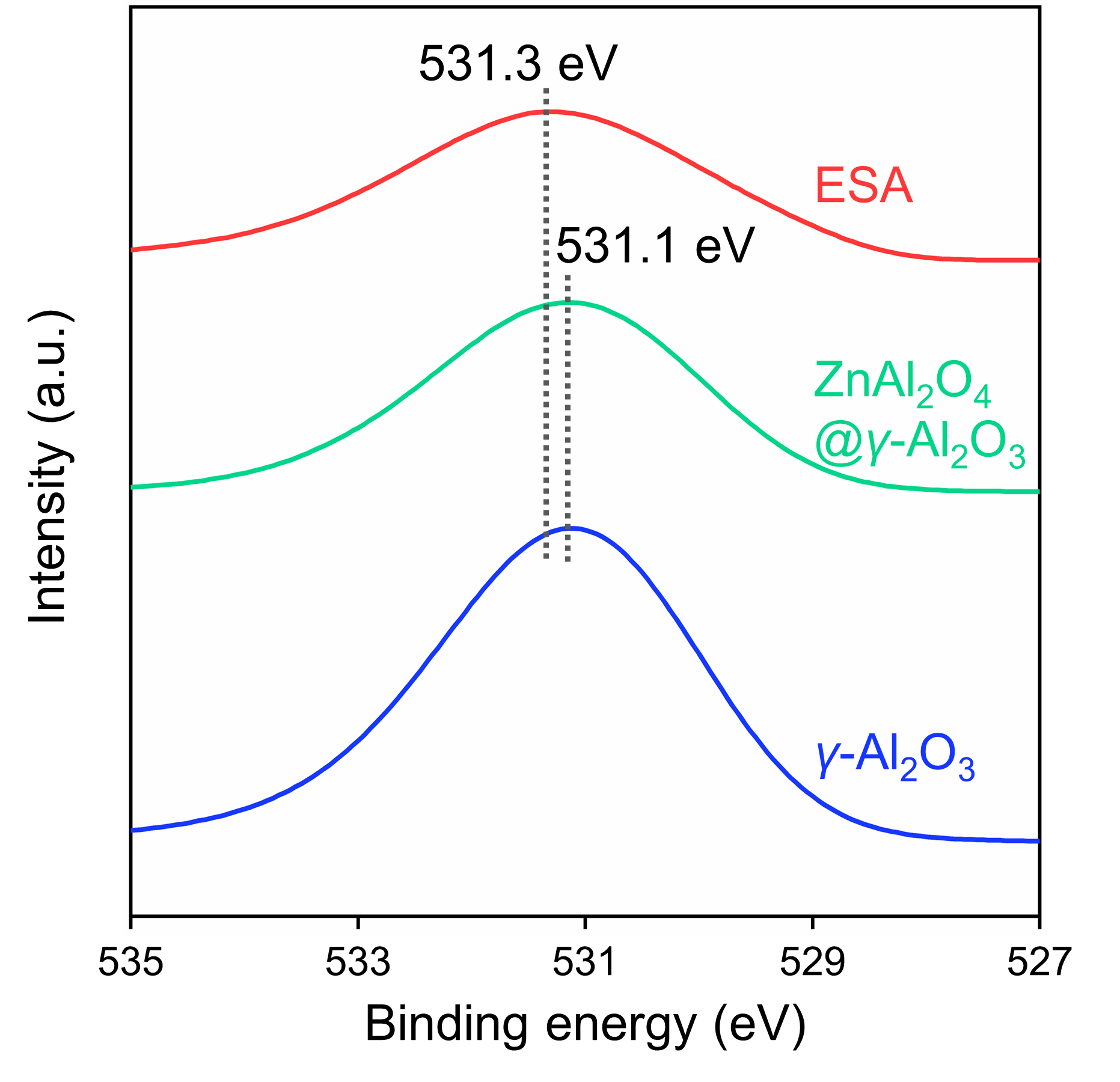
**

**Figure S13.** O 1s XPS spectra of the catalysts after He treatment at 873 K.


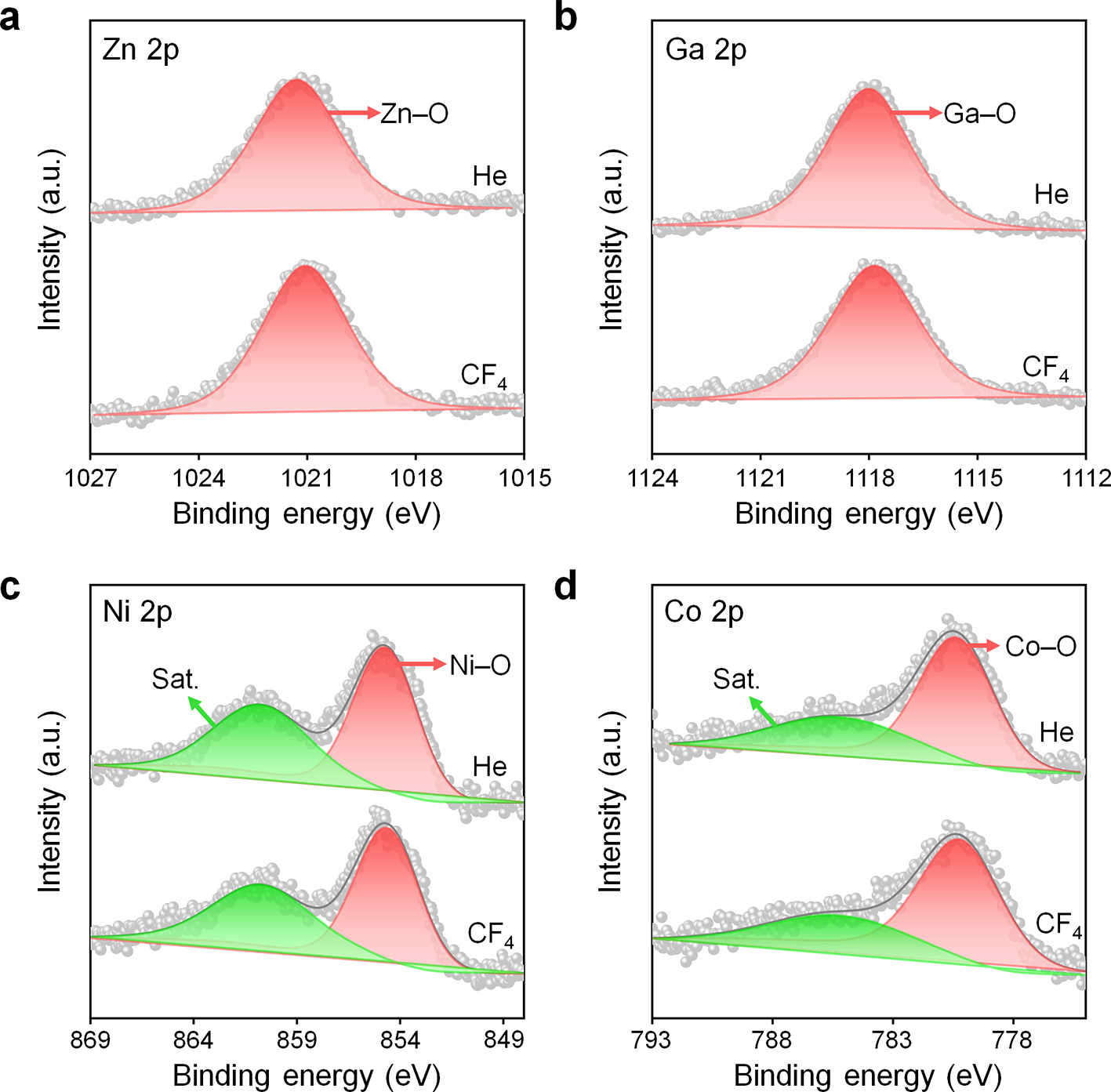


**Figure S14.** (a) Zn 2p, (b) Ga 2p, (c) Ni 2p, (d) Co 2p XPS spectra of ESA after He and CF_4_ treatments at 873 K. The high-binding-energy features in the Ni 2p and Co 2p regions correspond to intrinsic shake-up satellites (Sat.), which are commonly observed in such spectra.

**
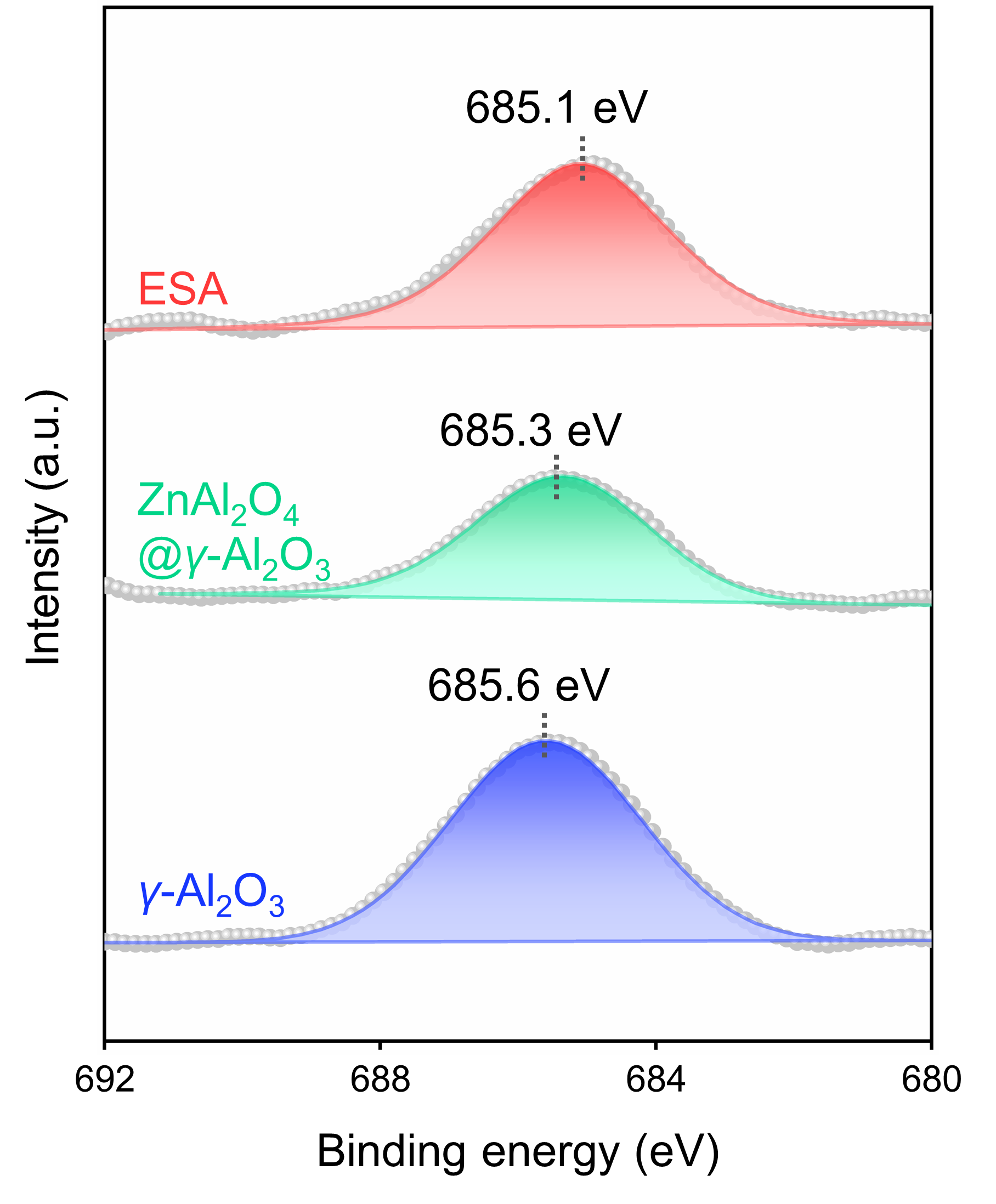
**

**Figure S15.** F 1s XPS spectra of the catalysts after CF_4_ treatment at 873 K.


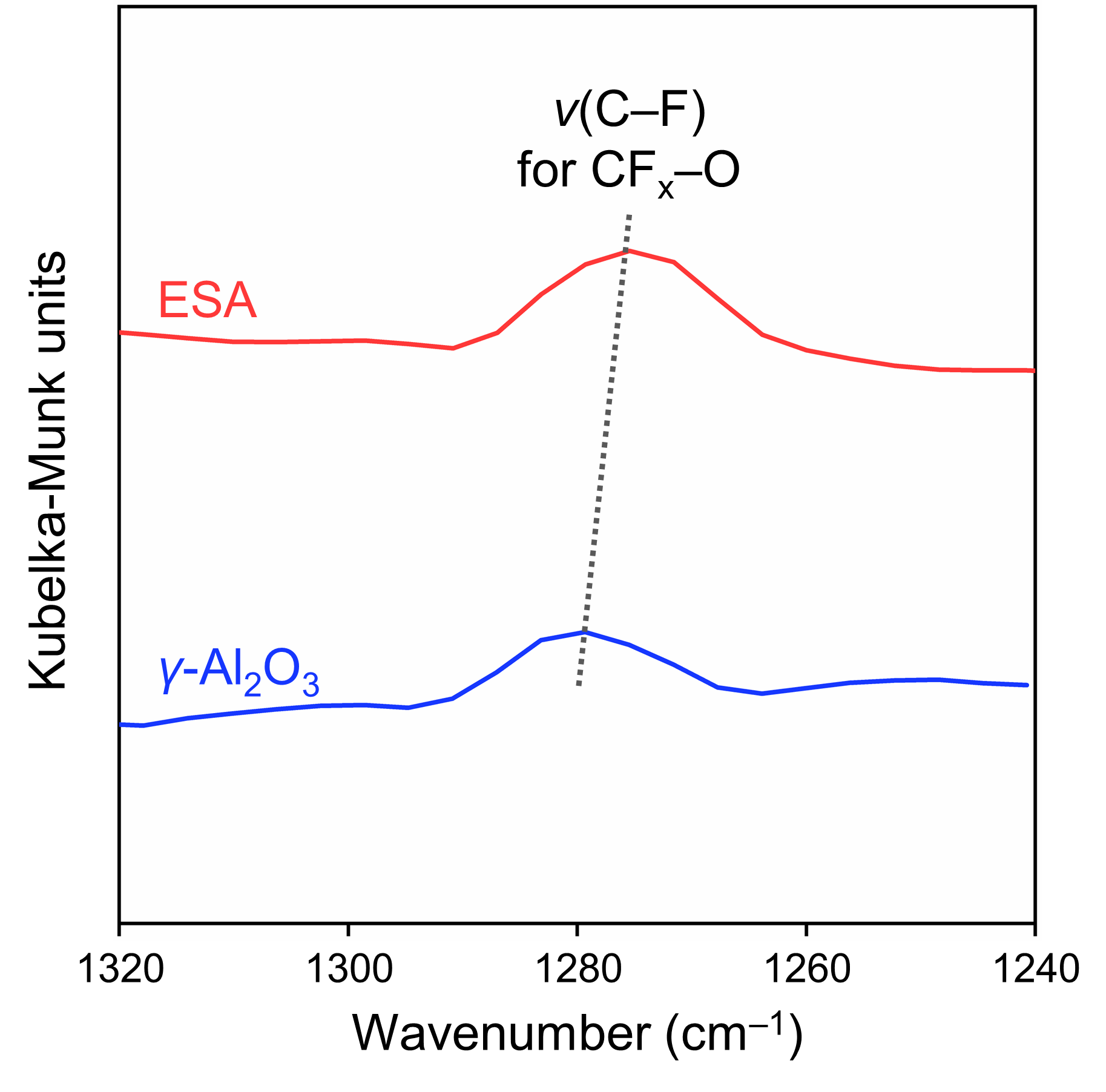


**Figure S16.** DRIFTS spectra of ESA and *γ*-Al_2_O_3_ measured after CF_4_ treatment at 773 K, subsequent cooling to 373 K, and He purging to eliminate gas-phase and weakly adsorbed CF_4_.

**
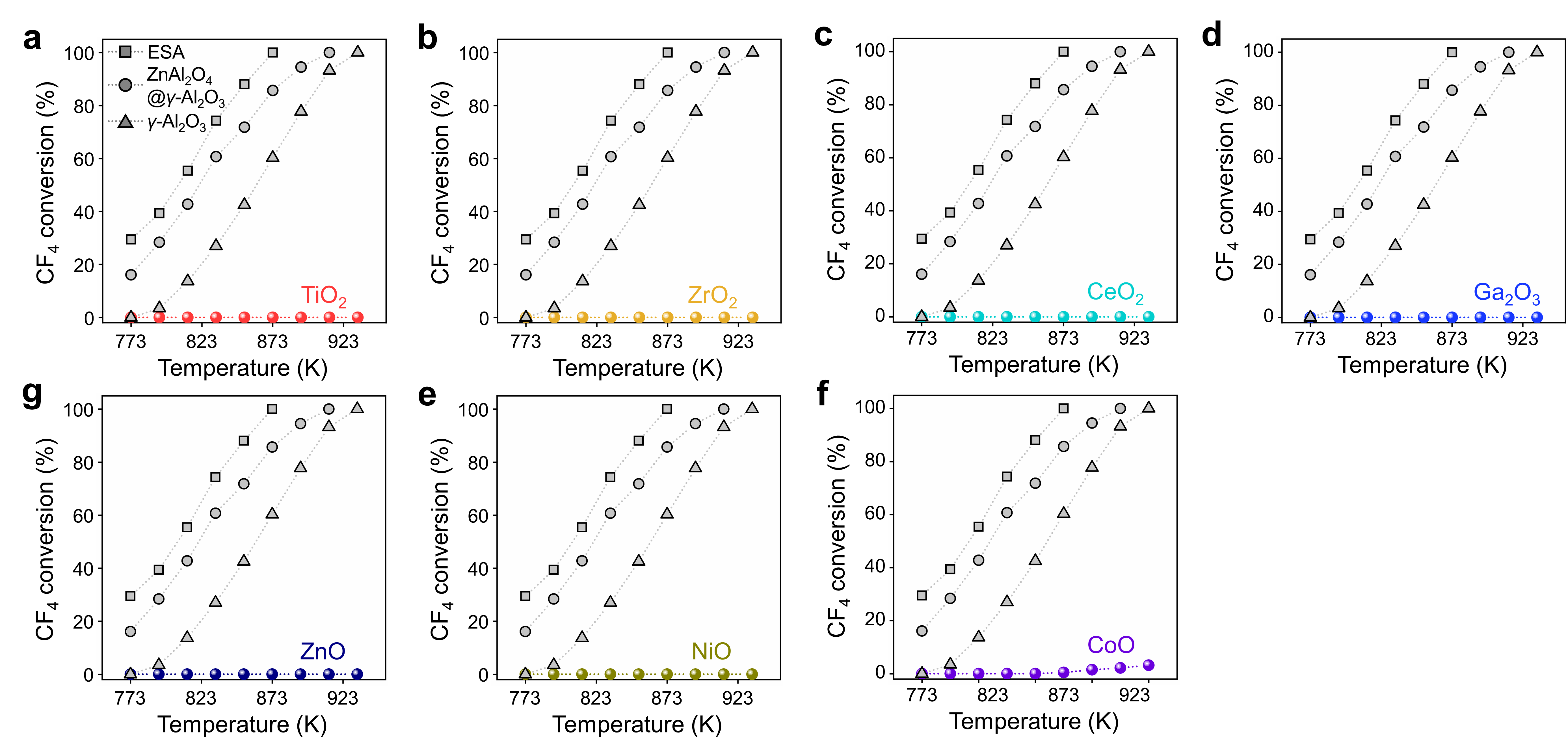
**

**Figure S17.** CF_4_ conversion as a function of reaction temperature in CF_4_ hydrolysis over single-metal oxide catalysts without Al (reaction conditions: 0.25 kPa CF_4_, 0.25 kPa Ar, and 12.7 kPa H_2_O in He balance; CF_4_-based WHSV = 0.01 h^–1^). For comparison, data for Al-containing catalysts are shown in gray.


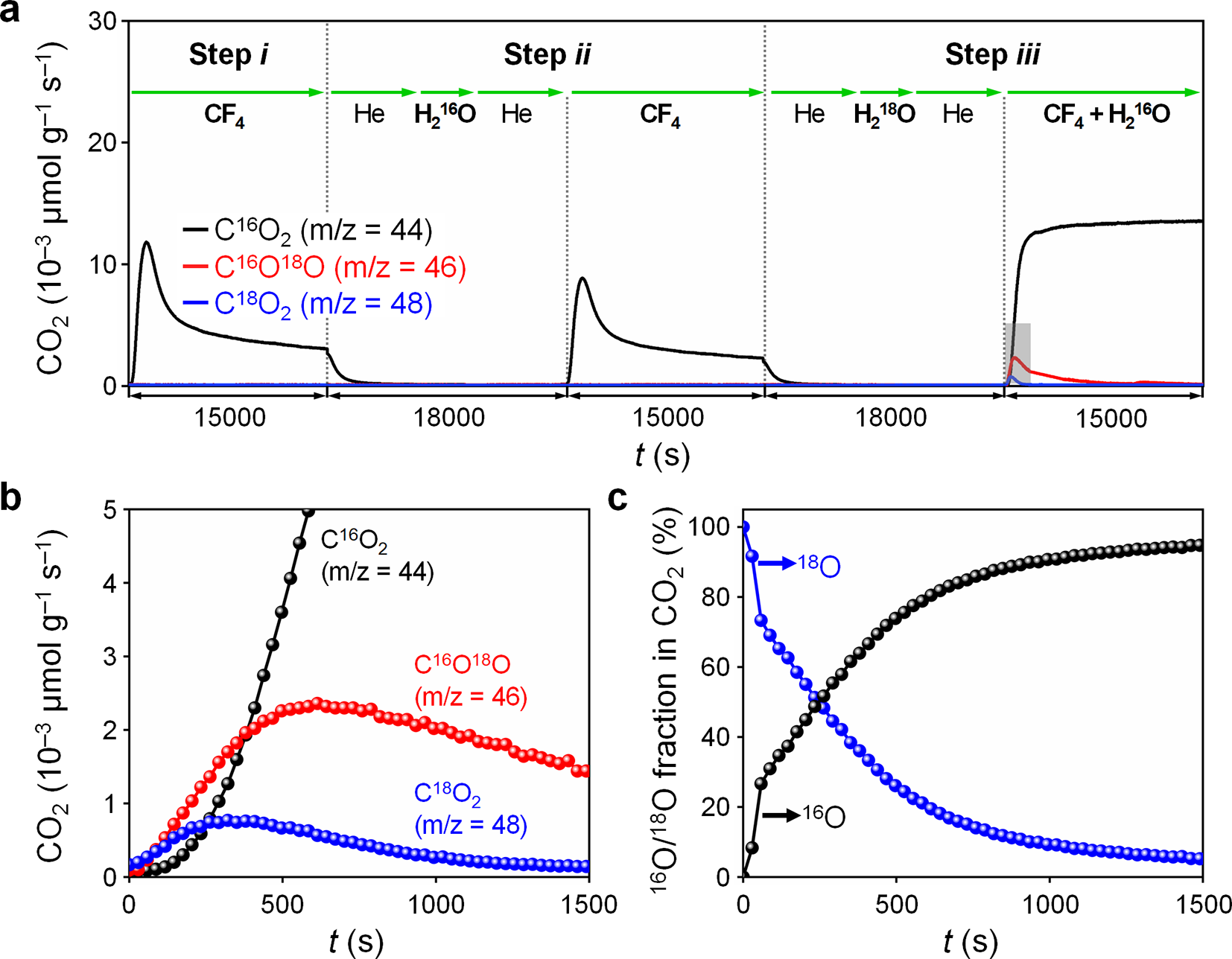


**Figure S18.** (a) The evolution of CO_2_ isotopologues during the sequential reaction experiment with the *γ*-Al_2_O_3_ catalyst at 873 K. Gas changes with time are summarized within the plot. (b) The CO_2_ isotopologue distribution immediately after introducing the CF_4_/H_2_^16^O mixture (magnified view of the shaded region in step *iii* of panel a). (c) The corresponding ^16^O/^18^O distribution within the CO_2_ product.


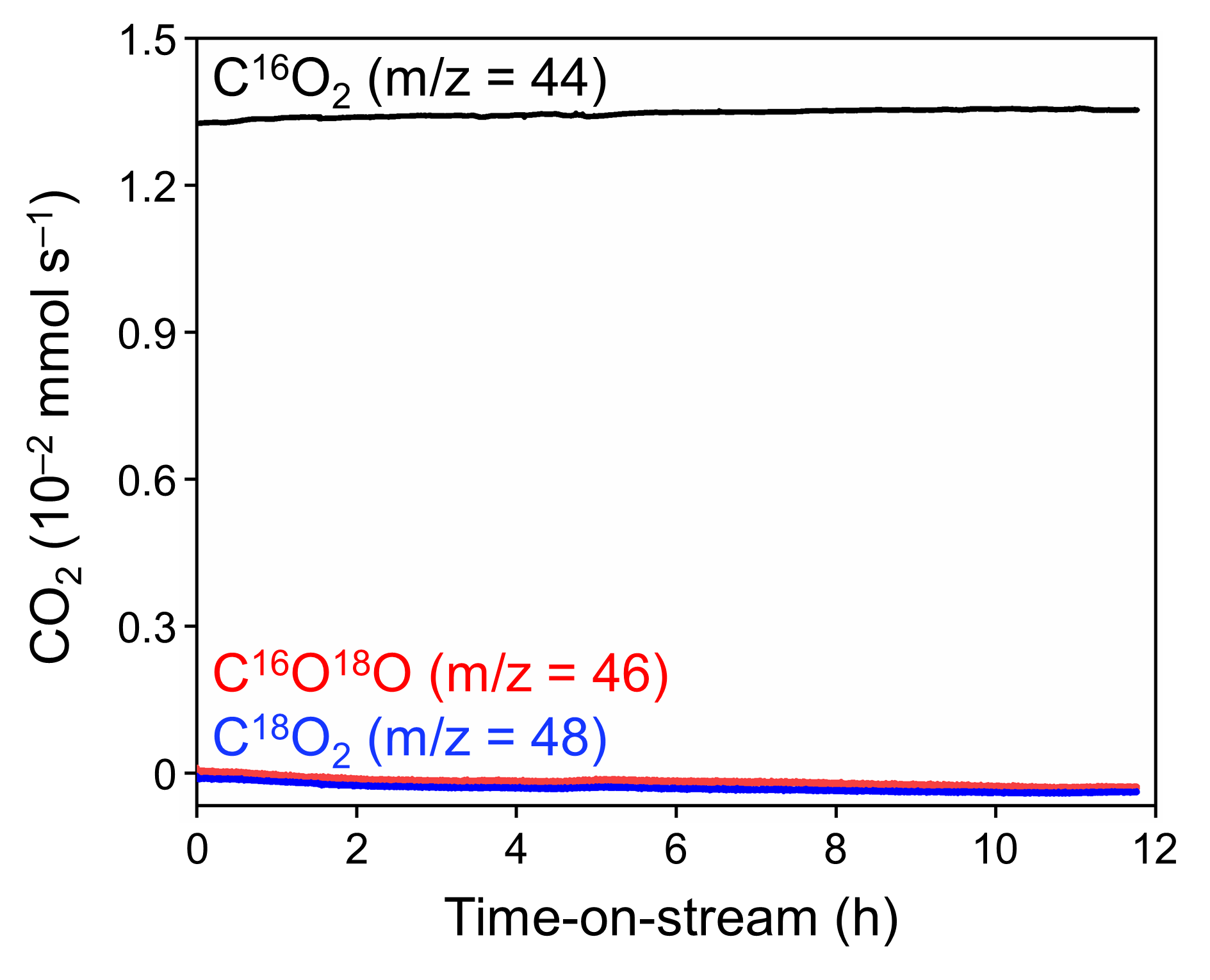
**Figure S19.** Blank experiment for testing gas-phase oxygen isotope exchange between C^16^O_2_ and H_2_^18^O at 873 K. The experiment was conducted in a catalyst-free reactor packed only with quartz wool under a flow of 33.3 kPa H_2_^18^O balanced with C^16^O_2_. No measurable formation of C^16^O^18^O or C^18^O_2_ was observed, indicating negligible gas-phase exchange.


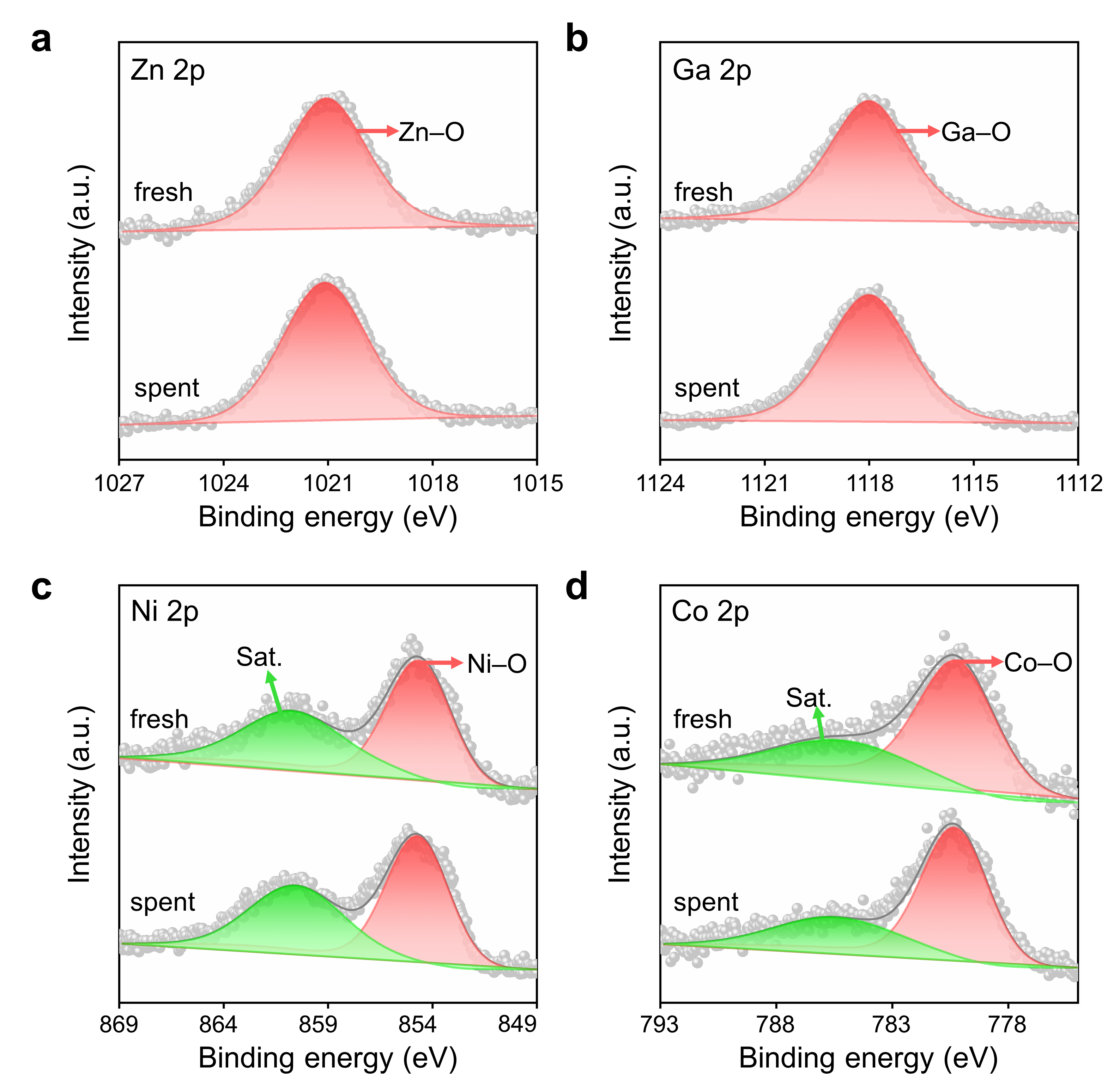


**Figure S20.** (a) Zn 2p, (b) Ga 2p, (c) Ni 2p, (d) Co 2p XPS spectra of fresh and spent ESA after 150 h of stability testing (reaction conditions: 1073 K, 0.67 kPa CF_4_, 0.67 kPa Ar, and 33 kPa H_2_O in He balance; CF_4_-based WHSV = 0.2 h^–1^). The high-binding-energy features in the Ni 2p and Co 2p regions correspond to intrinsic shake-up satellites (Sat.), which are commonly observed in such spectra.

**Figure S21.** Comparison of CF_4_ and C_2_F_6_ conversions as a function of reaction temperature over ESA during hydrolysis (reaction conditions: 0.25 kPa CF_4_ or C_2_F_6_, 0.25 kPa Ar, and 12.7 kPa H_2_O in He balance; CF_4_- or C_2_F_6_-based WHSV based on CF_4_ or C_2_F_6_ = 0.01 h^–1^).
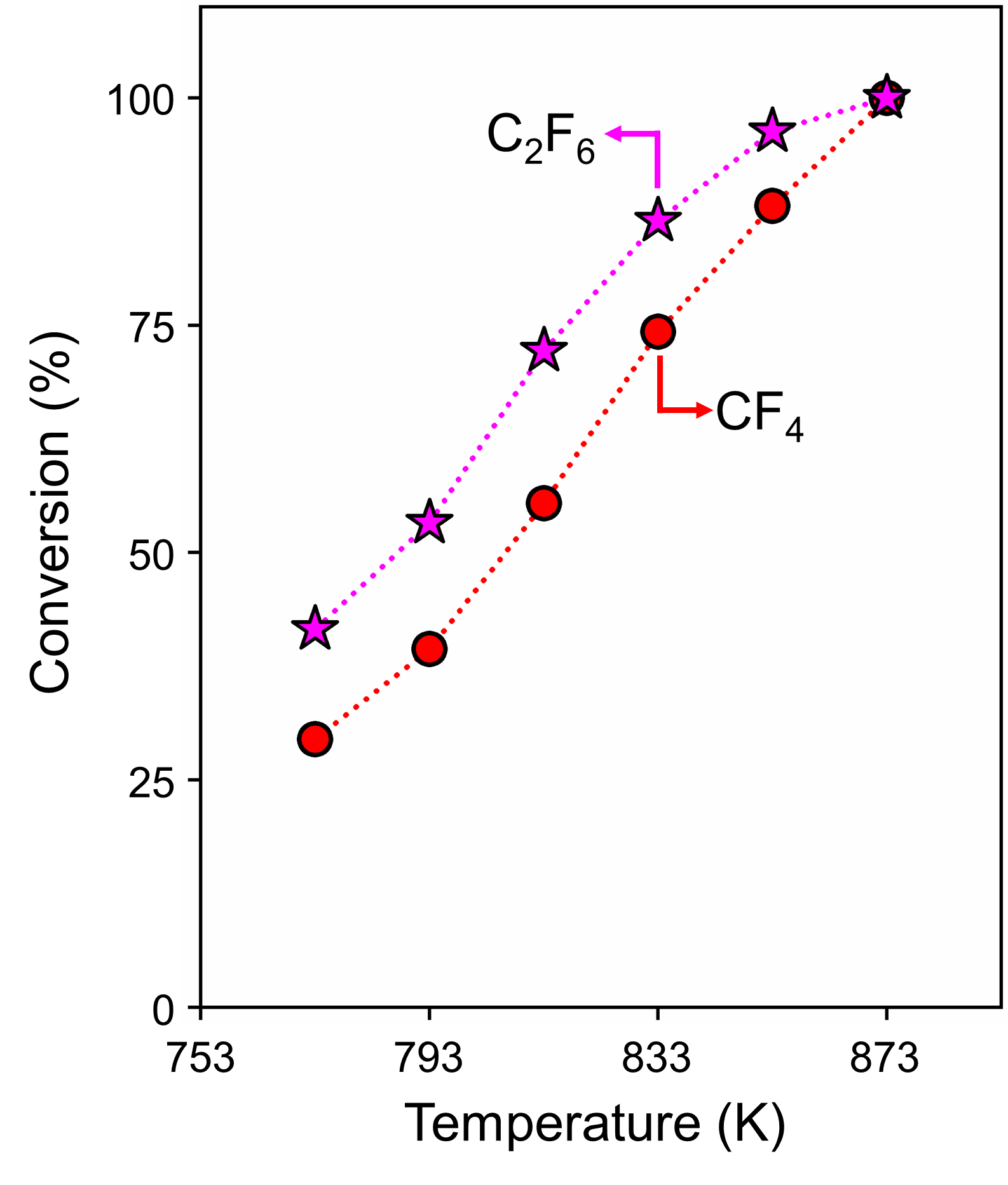


**Table S1**. Tolerance factor of candidate cations for spinel aluminate.

| Metals | Tolerance factor (*τ*) |
| --- | --- |
| Zn^2+^ | 0.84 |
| Ga^3+^ | 0.89 |
| Ni^2+^ | 0.86 |
| Co^2+^ | 0.85 |
| Mg^2+^ | 0.85 |

**Supplementary Note 1**

The spinel tolerance factor (*τ*) was calculated based on the normal spinel formula AB_2_X_4_, where A, B, and X correspond to the tetrahedral cation, octahedral cation, and anion, respectively. The following equation was used:

$$\tau(spinel tolerance factor)= \frac{\sqrt{3}(R_{B}+R_{X})}{2(R_{A}+R_{X})}$$

where *R*_A_, *R*_B_, and *R*_X_ are the ionic radii of the A-site cation, B-site cation, and X-site anion, respectively.^[1]^ In this study, *τ* was calculated for the corresponding aluminate spinel MAl_2_O_4_, where the candidate heterometal cation M occupies the A site, Al^3+^ occupies the B site, and O^2–^ occupies the X site. The ionic radius used for Al^3+^ in octahedral coordination was *R*_B_ = 0.54 Å. The ionic radius used for O^2–^ was *R*_X_ = 1.4 Å. *R*_A_ was assigned according to the tetrahedrally coordinated candidate cation: Zn^2+^ 0.60 Å, Ga^3+^ 0.47 Å, Ni^2+^ 0.55 Å, Co^2+^ 0.58 Å, and Mg^2+^ 0.57 Å. The calculated τ values are summarized in Table S1.

**Supplementary Note 2**

The configurational entropy (∆*S*_conf_) of ESA was calculated from the overall cation distribution, because the exact cation distribution over tetrahedral and octahedral sites cannot be uniquely determined from the present data. The following equation was used:

$${\Delta S}_{\mathrm{conf}}= -R\sum_{i} x_{i}\ln x_{i}$$

where *R* is the gas constant and *x_i_* is the molar fraction of each cation. For the ESA composition used in this study, the overall cation ratio is Al : Zn : Ga : Ni : Co = 8 : 1 : 1 : 1 : 1. Thus, the corresponding cation fractions are *x*_Al_ = 8/12 and *x*_Zn_ = *x*_Ga_ = *x*_Ni_ = *x*_Co_ = 1/12. The configurational entropy is therefore calculated as:

$${\Delta S}_{\mathrm{conf}}=-R\left( \frac{8}{12}\ln\frac{8}{12}+4\times\frac{1}{12}\ln\frac{1}{12} \right)=1.0986R \approx1.1R$$

This value is comparable to those reported for entropy-stabilized spinel and perovskite oxide systems, where ∆*S*_conf_ values in the range of approximately 1.0–1.3R have been used to describe entropy-stabilized oxide phases.^[2–5]^

**Table S2**. Elemental composition of ESA determined by EDS mapping.

|  | Zn/Al | Ga/Al | Ni/Al | Co/Al |
| --- | --- | --- | --- | --- |
| Atomic ratio | 0.12 | 0.13 | 0.11 | 0.12 |

**Table S3.** Physicochemical properties of Lewis acidic transition-metal oxides without Al.

| Sample | *S*_BET_^[a]^  [m^2^ g^–1^] | *V*_p_^[b]^ [cm^3^ g^–1^] | *n*_Lewis_^[c]^ [µmol g^–1^] |
| --- | --- | --- | --- |
| TiO_2_ | 10 | 0.021 | 76 |
| ZrO_2_ | 11 | 0.019 | 55 |
| CeO_2_ | 37 | 0.063 | 109 |
| Ga_2_O_3_ | 19 | 0.089 | 71 |
| ZnO | 11 | 0.018 | 10 |
| NiO | 1.7 | 0.034 | 8.2 |
| CoO | 135 | 0.27 | 15 |

**Table S4**. Comparison of CF_4_ hydrolysis conditions with those reported in representative literature studies.

[a] BET surface areas calculated from N_2_ adsorption isotherms in the *P*/*P*_0_ range of 0.05–0.15. [b] Total pore volumes determined at *P*/*P*_0_ = 0.95. [c] Lewis acid site concentrations determined by FT-IR spectroscopy after pyridine adsorption at 423 K.

| Reference | CF_4_ concentration (ppm) | CF_4_-based WHSV  (h^–1^) |
| --- | --- | --- |
| This work | 6700 | 0.20 |
| [6] | 10000 | 0.022 |
| [7] | 6700 | 0.12 |
| [8] | 12000 | 0.0086 |
| [9] | 2500 | 0.0090 |
| [10] | 2500 | 0.0090 |
| [11] | 2500 | 0.0090 |

**Supplementary References**

[1] Z. Song, Q. Liu, *Cryst. Growth Des.* **2020**, *20*, 2014–2018.

[2] C. Riley, N. Valdez, C. M. Smyth, R. Grant, B. Burnside, J. E. Park, S. Meserole, A. Benavidez, R. Craig, S. Porter, A. Delariva, A. Datye, M. Rodriguez, S. S. Chou, *J. Phys. Chem. C* **2023**, *127*, 11249–11259.

[3] E. Nidžović, B. Matović, P. Tatarko, N. Hosseini, O. Hanzel, L. Radovanović,  A. Dapčević, M. P. Đorđević, *J. Eur. Ceram. Soc.* **2025**, *45*, 117582.

[4] L. Shen, Z. Du, Y. Zhang, X. Dong, H. Zhao, *Appl. Catal. B: Environ.* **2021**, *295*, 120264.

[5] D. Zhang, J. Park, B. Xu, C. Liu, W. Li, X. Liu, Y. Qi, J. Luo, *Dalton Trans.* **2023**, *52*, 1082–1088.

[6] Z. M. El-Bahy, R. Ohnishi, M. Ichikawa, *Appl. Catal. B: Environ.* **2003**, *40*, 81–91.

[7] Z. M. El-Bahy, R. Ohnishi, M. Ichikawa, *Catal. Today* **2004**, *90*, 283–290.

[8] J. Y. Jeon, X. F. Xu, M. H. Choi, H. Y. Kim, Y. K. Park, *Chem. Commun.* **2003**, 1244–1245.

[9] W. Luo, K. Liu, T. Luo, J. Fu, H. Zhang, C. Ma, T. S. Chan, C. W. Kao, Z. Lin, L. Chai, M. L. Coote, M. Liu, *J. Am. Chem. Soc.* **2025**, *147*, 7391–7399.

[10] X. Wang, J. Fu, H. Zhang, J. Zheng, H. Chen, K. Liu, L. Jing, X. Xing, Z. Lin, L. Chai, M. Liu, *Environ. Sci. Technol.* **2025**, *59*, 3309–3315.

[11] H. Chen, H. Zhang, X. Wang, W. Luo, J. Zheng, K. Liu, J. Fu, H. Li, Z. Lin, L. Chai, N. Hu, M. Liu, *Environ. Sci.: Nano* **2025**, *12*, 3530–3538.
